# A comprehensive review of *Rubia cordifolia* L.: Traditional uses, phytochemistry, pharmacological activities, and clinical applications

**DOI:** 10.3389/fphar.2022.965390

**Published:** 2022-09-09

**Authors:** Min Wen, Qi Chen, Wang Chen, Jing Yang, Xiaogang Zhou, Chunxiang Zhang, Anguo Wu, Jia Lai, Jianping Chen, Qibing Mei, Shuo Yang, Cai Lan, Jianming Wu, Feihong Huang, Long Wang

**Affiliations:** ^1^ School of Pharmacy, Southwest Medical University, Luzhou, China; ^2^ Department of Endocrinology and Metabolism, The Affiliated Hospital of Southwest Medical University, Luzhou, China; ^3^ Institute of Cardiovascular Research, The Key Laboratory of Medical Electrophysiology, Ministry of Education of China, Medical Key Laboratory for Drug Discovery and Druggability Evaluation of Sichuan Province, Luzhou, China; ^4^ School of Chinese Medicine, LKS Faculty of Medicine, The University of Hong Kong, Pok Fu Lam, Hong Kong SAR, China

**Keywords:** *Rubia cordifolia* L., traditional uses, pharmacological activities, phytochemistry, clinical application

## Abstract

*Rubia cordifolia* (family: Rubiaceae) L (*R. cordifolia*) is a perennial botanical drug climbing vine. As the main part of the traditional Chinese medicine, the rhizome has a long history. A great number of literary studies have reported that it can be used for the improvement of blood circulation, hemostasis, activation of collaterals, etc. When it comes to the wide application of *R. cordifolia* in traditional medicine, we systematically review its traditional uses, phytochemistry and pharmacological effects. Literatures were systematically searched using several scientific databases, including *China National Knowledge Infrastructure* (CNKI), *Baidu Scholar*, *PubMed*, *Web of Science*, and other professional websites. Kew Botanical Garden and the *iPlant* were used for obtaining the scientific names and plant images of *R. cordifolia*. In addition, other information was also gathered from books including traditional Chinese herbal medicine, *the Chinese Pharmacopoeia*, and *Chinese Materia Medica*. So far, many prescriptions containing *R. cordifolia* have been widely used in the clinical treatment of abnormal uterine bleeding, primary dysmenorrhea and other gynecological diseases, allergic purpura, renal hemorrhage and other diseases. The phytochemistry studies have reported that more than 100 compounds are found in *R. cordifolia*, such as bicyclic peptides, terpenes, polysaccharides, trace elements, flavonoids, and quinones. Among them, quinones and peptides are the types of components with the highest contents in *R. cordifolia*. The modern pharmacological studies have revealed that *R. cordifolia* and its derived components have anti-tumor, anti-oxidative, anti-platelet aggregation, and anti-inflammatory effects. However, most studies are preclinical. The pharmacological mechanism of *R. cordifolia* has not been thoroughly studied. In addition, there are few pharmacokinetic and toxicity studies of *R. cordifolia*, therefore the clinical safety data for *R. cordifolia* is lacking. To sum up, this review for the first time summarizes a systemic and integrated traditional uses, chemical compositions, pharmacological actions and clinical applications of *R. cordifolia*, which provides the novel and full-scale insight for the drug development, medicinal value, and application of *R. cordifolia* in the future.

## Introduction

Plants are invaluable reservoir for the discovery of new drugs, which have been used for medicinal purposes across history and cultures. As the World Health Organization (WHO) stated, approximately 80% of people all over the world relies heavily on botanical medicine for their primary health care (Ekor, 2014). Traditional Chinese medicine (TCM) are a treasure trove of drugs, which have been widely spread and applied in more than 100 countries for the treatment of diverse diseases (Wang et al., 2016). With the Coronavirus disease (COVID-19) pandemic quickly spreads across the whole world, TCM have become a key component in the treatment of COVID-19 and play an irreplaceable role in the treatment of SARS-CoV-2 infection (Wang and Yang, 2021). TCM have the unique advantage of whole course and all-round treatment that could improve symptoms, early prevention, early treatment and reduce mortality, and reduce the occurrence and recurrence of complications, which have broad application prospect (Dai et al., 2020).

The Rubiaceae family consists of about 450 genera and 6,500 species, including trees, shrubs and herbs. Rubia has about 60 species, of which *R.* co*rdifolia* L. (Rubiaceae) is a perennial herbaceous climbing plant with long, cylindrical and red roots (Adwankar et al., 1980; Dengre et al., 1993). *R. cordifolia* is widely distributed in Africa, tropical Asia, India, Malaysia, China, Japan, and tropical Australia (Adwankar et al., 1980). In China, the root of *R. cordifolia* is known as *Qiancaogen* and *Chien-tsao*, which is widely distributed in most areas of China, especially shaanxi, Henan, Anhui, Hebei, Shandong, Jiangsu and Zhejiang provinces (Dengre et al., 1993). In Chinese medicine, *R. cordifolia* is bitter with cold property, which is able to eliminate pathogenic heat from blood, remove blood stasis and dredging collaterals, and promote hemostasis. It is mainly used as a hemostatic agent in *folk* medicine to treat hematemesis, metrorrhagia, epistaxis, wounds, injuries and strains (Xu et al., 2002; Li et al., 2016). In addition, the aerial part of the *R. cordifolia* plant, known locally as “*Guoshan dragon*” in Shaanxi province in China, has been used to treat diarrhea more than a century. The plant water decoction can be taken orally to treat diarrhea, which can also be used externally for foot bath when children are too young to take it. *R. cordifolia* is also a main ingredient of formula named “*Er-Xie-Ting granule*”, which is used to treat acute infantile diarrhea in China (Shi et al., 2014). In India, *R. cordifolia* is often known as Madder or India Madder. Locals in India call it “Manjistha”. Its dried samples are sold in the market under the name “Manjith”. *R. cordifolia* is very common at high altitudes, Mahabaleswar, Amboli and Maharastra state in India (Adwankar et al., 1980; Siril, 2013). This drug is used to treat *Shi Feng Bi* (rheumatism), menstrual pain, urinary system diseases, dropsy, paralysis, amenorrhea and jaundice in India (Tripathi et al., 1993; Adams et al., 2009). In Ayurveda, *R. cordifolia* has been used as a coloring agent for medicinal oils, and applied externally to inflamed areas, ulcers and fractures. *R. cordifolia* root has been treated various chronic inflammations (Deshkar et al., 2008; Patil et al., 2009). A paste made from honey is applied to the skin to remove brown spots, freckles and other skin discoloration and is used to promote wound healing (Adwankar et al., 1980; Pawar et al., 2009). In Korea, *R. cordifolia* is widely utilized as a traditional remedy for dysmenorrhea, arthritis, rheumatism, hematorrhea and urinary disorders (Do et al., 2013). In Unani medicine, the dark red root of *R. cordifolia* is used to invigorate spleen and soothing liver, dysmenorrhea, diuresis, paralysis, jaundice, amenorrhea, skin disorders of many varieties, renal stone and blood detoxification. In Uganda medicine, traditional healers use the drug to treat tuberculosis cases. In the Philippines medicine, root decoction of *R. cordifolia* is used to treat urinary tract disorders (Patil et al., 2009). In traditional Asian medicine, the roots of *R. cordifolia* have become an important drug for the treatment of abnormal uterine bleeding (AUB), purpura lupus erythematosus, hemorrhage syndrome, arthritis, kidney stones, hemostasis, hysteresis, and psoriasis (Chang et al., 2000; Basu et al., 2005; Tse et al., 2007; Son et al., 2008; Wang K. et al., 2020). In ancient Europe, *R. cordifolia* is one of the most commonly used dyes, which is used for dyeing wool, silk, linen, cotton fabrics, as well as basket-making material. The roots of the plant are also one of important ingredients in recipes of red inks. Alizarin is the main component of dyes (Wu and Yang, 2016). On the whole, *R. cordifolia* is the first batch of plants known to have commercial and medicinal value in the world (Feng et al., 2021). The drug is highly valued for its pharmacological, cardioprotective and industrial approach. Except for its high medicinal value, the drug is also an important source of natural dye used by many flavours and pharmaceutical industries (Pankaj and A., 2013bib_Pankaj_and_Ajay_2013bib_Pankaj_and_Ajay_2013bib_Pankaj_and_Ajay_2013). *R. tinctorum* L. another member of Rubiaceae family, is also a well-known traditional medicinal plant, which is native to Europe, west of Asia and Africa. Like *R.* co*rdifolia*, *R. tinctorum* L. has been used as a dye for over 2,000 years (Kalyoncu et al., 2006). In Morocco, *R. tinctorum* L. has been usded to treat renal disease, cardiac disease, hypertension and diarrhoea (Jouad et al., 2001; Eddouks et al., 2002; Karim et al., 2010). *R. tinctorum* L. is recorded in the 14th edition of the modern Russian Pharmacopoeia with a diuretic effect (Shikov et al., 2021). Due to the genotoxic activity and oncogenic potential of *R. tinctorum* L. Commission of the European Communities has considered *R. tinctorum* L. as a plant with serious risks (Cpmp, 2004).

Studies have found that *R. cordifolia* is rich in more than 100 compounds, mainly including anthraquinones, naphthoquinones, anthraquinone glycosides, naphthoquinone glycosides, bicyclic hexapeptides, triterpenoids and polysaccharides (Son et al., 2008; Itokawa et al., 1993; Rao et al., 2006; Gao et al., 2016; Natarajan et al., 2019). *R. cordifolia* has multiple pharmacological activities, such as neuroprotective, anti-tumor, antibacterial, anti-inflammatory, anti-oxidant, and immunosuppressive effects (Lee et al., 2008a; Son et al., 2008; Shilpa et al., 2012b; Shen et al., 2018; Balachandran et al., 2021).

**FIGURE 1:**
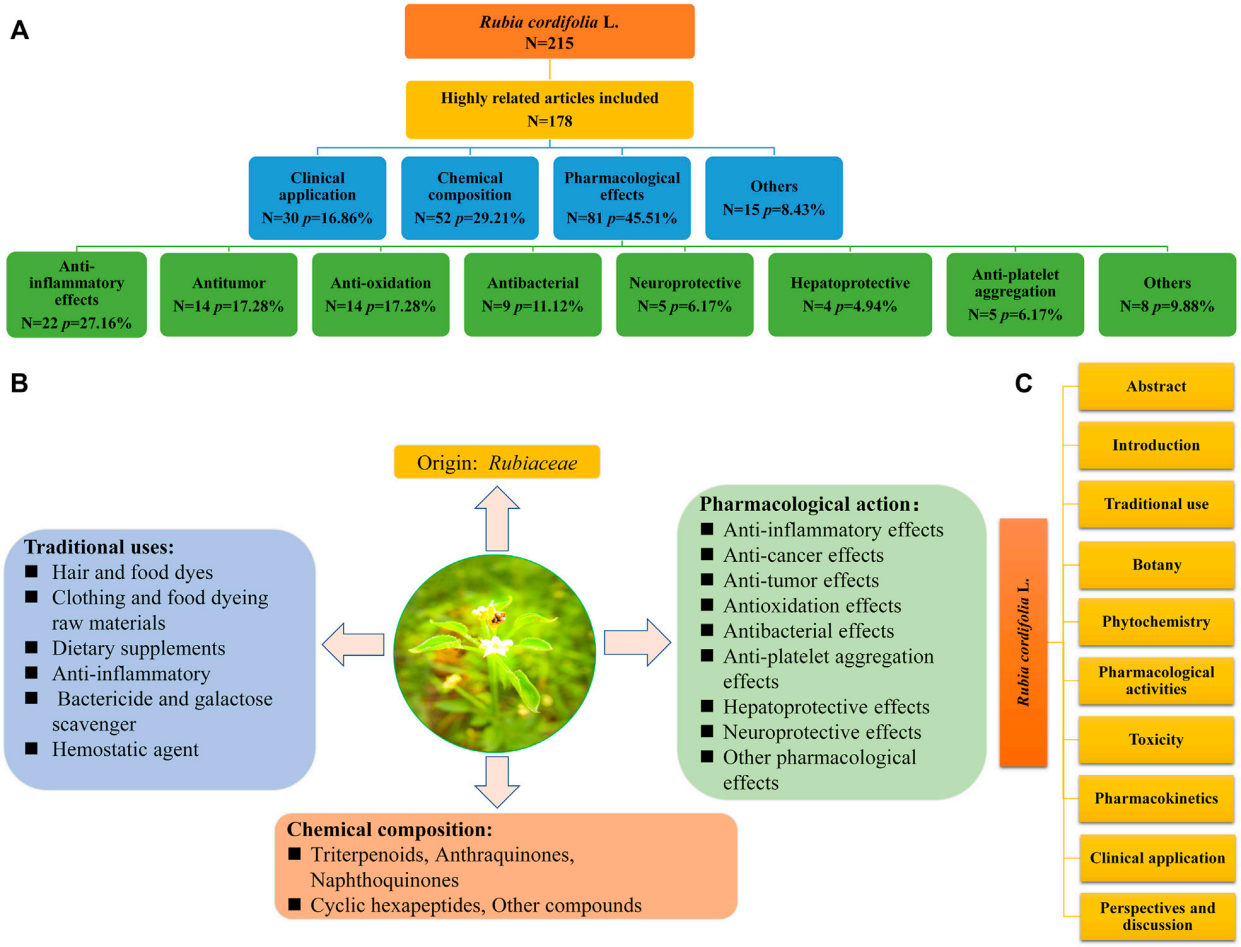
Reports on *R. cordifolia* were collected from PubMed database. Systematic classification of retrieved literatures *R. cordifolia* **(A)**. Origin, traditional uses, pharmacological effects, and chemical compositions of *R. cordifolia* **(B)**. Classification and analyzation of the content of the articles **(C)**.

In present review, the keyword *Rubia cordifolia* L. was used to search the literatures from *Google scholar*, *Web of Science*, *and PubMed*. The latest publication time of these literatures were up to May 2022. We had analyzed these articles and made a classification summary. By document retrieval, we summarized the total number and proportion of articles on *R. cordifolia*, such as the total number and proportion of clinical application, pharmacological action, or active ingredients (Figure 1A). Meanwhile, we also introduced the origin, traditional application, chemical compounds and pharmacological effects of *R. cordifolia* (Figure 1B). Then the overall framework of this article was summarized (Figure 1C). Finally, we hope this review can provide an integrated insight for the drug development, medicinal value, and application of *R. cordifolia* in the future.

## Traditional use

In view of the widespread distribution and application of *R. cordifolia* in the world, there are differences in application in different countries and regions. According to historical records, *R. cordifolia* was used as an important natural dye for hair, food and clothing (Ni et al., 2022; Yusuf et al., 2013). It was widely used in ancient China and India with a long history. As early as 2000 Before Christ (B.C.), it had been used as a folk medicine to treat wounds, ulcers, skin diseases, rheumatic, and inflammatory-related diseases. As an important traditional medicine, *R. cordifolia* was firstly recorded in the *Huangdi Neijing* (Li et al., 2018), also recorded in some Chinese classical prescriptions, such as *Bencaijing Jizhu*, *52 Neijing*, *Shu Bencao*, *History of Chinese Traditional Medicine*, and so on. Nowadays, *R. cordifolia* is officially listed in the *Chinese Pharmacopoeia*.

The theory of TCM is the understanding of the general laws of drugs, involving four odors and five flavors, ascending and descending, channel tropism, toxicity and side effects (Zhong et al., 2019). In the field of TCM, the durg *R. cordifolia* is benefit for cooling blood, hemostasis, removing blood stasis, and relieving pain, with bitter taste, cold property and distribution to liver meridian. This traditional efficacy of *R. cordifolia* can be translated into modern pharmacological effects such as anti-inflammatory, anti-coagulant and anti-platelet activities as well as spasmolysis (Tripathi et al., 1993; Pawar et al., 2011). *R. cordifolia* with different processing methods has different curative effects (Li et al., 2018). For example, the raw *R. cordifolia* product has the effect of promoting blood circulation, removing blood stasis, clearing heat and cooling blood (Chen, 2013). However, after frying with charcoal, the coldness of *R. cordifolia* will be weakened, property and taste will be astringent, and it is mainly applied to stop bleeding (Gao et al., 2020). Based on the theory of TCM, *R. cordifolia* belongs to the liver meridian and has anti-liver cancer effect (Li et al., 2016). Furthermore, *R. cordifolia* is traditionally used as an a ntioxidation, bactericide, and galactose scavenger, and is also widely used in the pharmaceutical industry (Shilpa et al., 2012a). In ancient Chinese classic prescriptions and some minority medical books, *R. cordifolia* can be combined with other Chinese medicines to treat a variety of symptoms. For instance, combination *Cirsium arvense* var, *Gardenia jasminoides* Ellis with *R. cordifolia* can enhance the hemostatic effect (Wang et al., 2021). Moreover, joint prescription with *Sanguisorba officinalis* L. is able to treat the symptoms of intestinal dryness and bleeding. When combined with *Salvia miltiorrhiza* Bunge and *Angelica sinensis* (Oliv.) Diels, it can effectively improve the symptoms of dysmenorrhea (Li et al., 2009), so as to achieve the effect of multi-drug compatibility in treating various diseases **(** Table 1 **)**.

**TABLE 1:** Clinical application of *R. cordifolia* in traditional prescriptions.

Classic prescriptions	Composition of medicines	Symptoms of treatment	Type and number of participants	References
*Puji Benshifang*	*R. cordifolia*, *Artemisia argyi Levl.et* Vant, *Prunus mume* (Sieb.) Sieb.et Zuce	Treatment of nosebleeds	50 patients with nosebleeds. (age range, 14–76 years old)	(Li, 2000; Meng, 2010)
*Qixiongwan*	*R. cordifolia*, *Aconitum kusnezoffii* Reichb, *Moschus*, *Terminalia chebula* Retz, *Ruta graveolens* L, *Mercury* (II) *Sulfide*, *O. chiliophyllaRoyle*	Treatment of *Mycoplasma pneumoniae* pneumonia	21 patients with positive serum *mycoplasma* antibody test. (age range, 3–39 years old)	Du, (2002)
*Shengjie zonglu*	*R. cordifolia*, *Glycyrrhiza uralensis* Fisch	Treatment of vomiting blood, antipyretic and thirst, detoxification	60 patients (36 female and 24 male) with fever. (age range, 15–60 years old)	(Gupta et al., 2008; Li and Meng, 2018; Wang et al., 2022)
*Sanhongtang*	*R. cordifolia*, *Eriobotrya japonica* Thunb, *Laccifer lacca* Kerr	Treatment of kidney damage, lung heat, cough, blood in sputum, bladder heat, dysuria and frequent urination	Not available	Larga et al. (2021)
*Sanwei Chafenliaotang*	*R. cordifolia*, *Ophiopogon japonicus* (Linn. f.) Ker-Gawl, *Polygonum divaricatum* L	Treatment of fever in the lungs and tingling in the intestines	Not available	Liu et al. (2021)
*Sibu Yidian*	*R. cordifolia*, *Terminalia chebula* Retz. *Laccifer lacca* Kerr. *Symplocos caudata *Wall	Treatment of kidney and enteric fever	Not available	Niang et al. (2021)
*Shisanwei Ximingwan*	*R. cordifolia*, *Patrinia scabiosaefolia*, *Erminalia chebula* Retz, *Laccifer lacca* Kerr, *Caesalpinia decapetala *(Roth) Alston, Mangifera indica L, Syzygium jambos (L.) Alston, Shanfanshi, Cupressus funebris Endl, *Amomum cardamon*, *Herpetospermum pedunculosum* (Ser.) C. B, *veronica eriogyne* H. Wink	Treatment of chronic prostatitis	93 patients with chronic prostatitis. (age range, 30–60 years old)	(Zhou, 2017; Liu et al., 2020)
*Tangyao Jingyanfang*	*R. cordifolia*, *Asini Corii* Colla, *Platycladus orientalis* (L.) Franco, *Platycladus orientalis* (L.) Franco	Pentecostal acts for healing women	181 patients with blood deficiency. (age range, 18–60 years old)	(Li and Meng, 2018; Zhang et al., 2021)
*Taiping Shenghuifang*	*R. cordifolia*, *Punica granatum* L	Treatment of prolapse of the anus and menorrhagia	48 female patients. (age range, 15–50 years old)	(Shen, 2013; Bryant-Smith et al., 2018)
*Yiashen Shiqiweiwan*	*R. cordifolia, Terminalia chebula* Retz, *Moschus*, *Aconitum kusnezoffii* Reichb, *Acorus tatarinowii*, *Aucklandia lappa* Decne, *Haliotis discus* hannai, *Mercury* (II) *Sulfide*, *Pulvis billis* bovis, *Ruta graveolens *L. *Canavalia gladiata* (Jacq.) DC, *Carthamus tinctorius* L, *Eriobotrya japonica* Thunb, *Xiangmo*, *Amomum kravanh Pierre ex* Gagnep, *Laccifer lacca* Kerr. *Dashua* Jihua	Treatment of chronic epididymal and spermatic hydrocele	324 patients with chronic epididymis. (age range, 7–89 years old)	(Wu, 2008a; Wu, 2008b; Wang, 2013)
			32 patients with hydrocele of the spermatic cord. (age range, 3–12 years old)	

Not available: The combination of *R. cordifolia* and other drugs for the treatment of some diseases has been recorded in traditional Chinese prescriptions, but these clinical data have not been reported in any literatures.

## Botany

**FIGURE 2:**
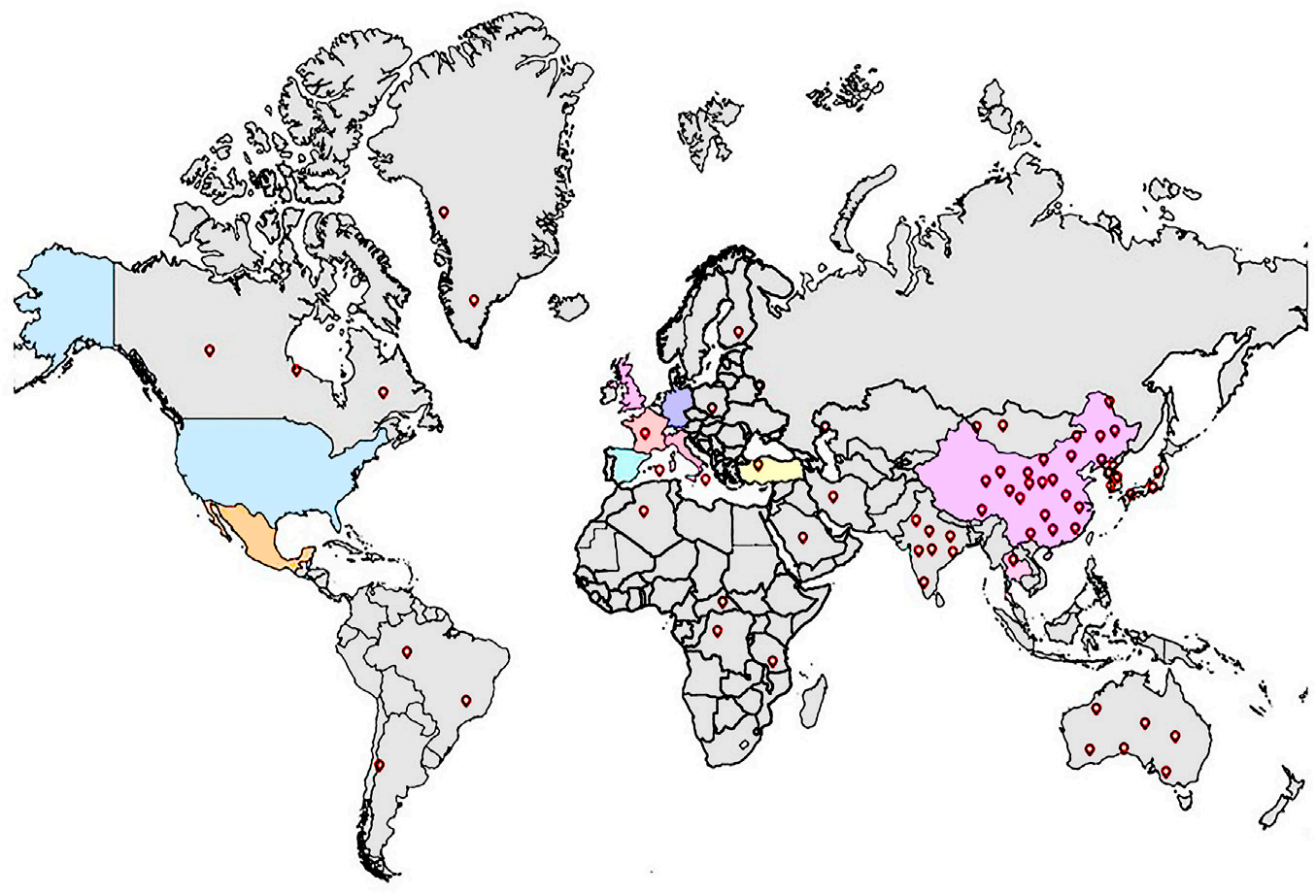
Geographical distribution of *Rubia cordifolia* L (world map from: https://www.onlinedown.net/soft/197029.htm) in the world (The red surface symbol map shows the distribution area of *R. cordifolia*).

*R. cordifolia* is distributed all over the world and is extremely widespread in many provinces of China, such as Shanxi, Henan, Anhui, Hebei, Shandong, Hubei, Jiangsu, Zhejiang, etc. (Figure 2). The global distribution data of this plant comes from the websites: iplant (http://ppbc.iplant.cn/) and Kew Botanical Garden (https://powo.science.kew.org/). Among them, Weinan city in Shanxi and Songxian city in Henan have the highest yields and the best quality. *R. cordifolia* is a perennial herbaceous climbing vine, scrambling, climbing or creeping plant. The root is brown or red. The stem is slender and rough, and the base is lignified. Its branching stems are 0.3–6 m long with brittle stems, strong curved prickles on the four ribs, or fully pubescent, or at least hairy below the nodes. Its roots are usually woody.

**FIGURE 3:**
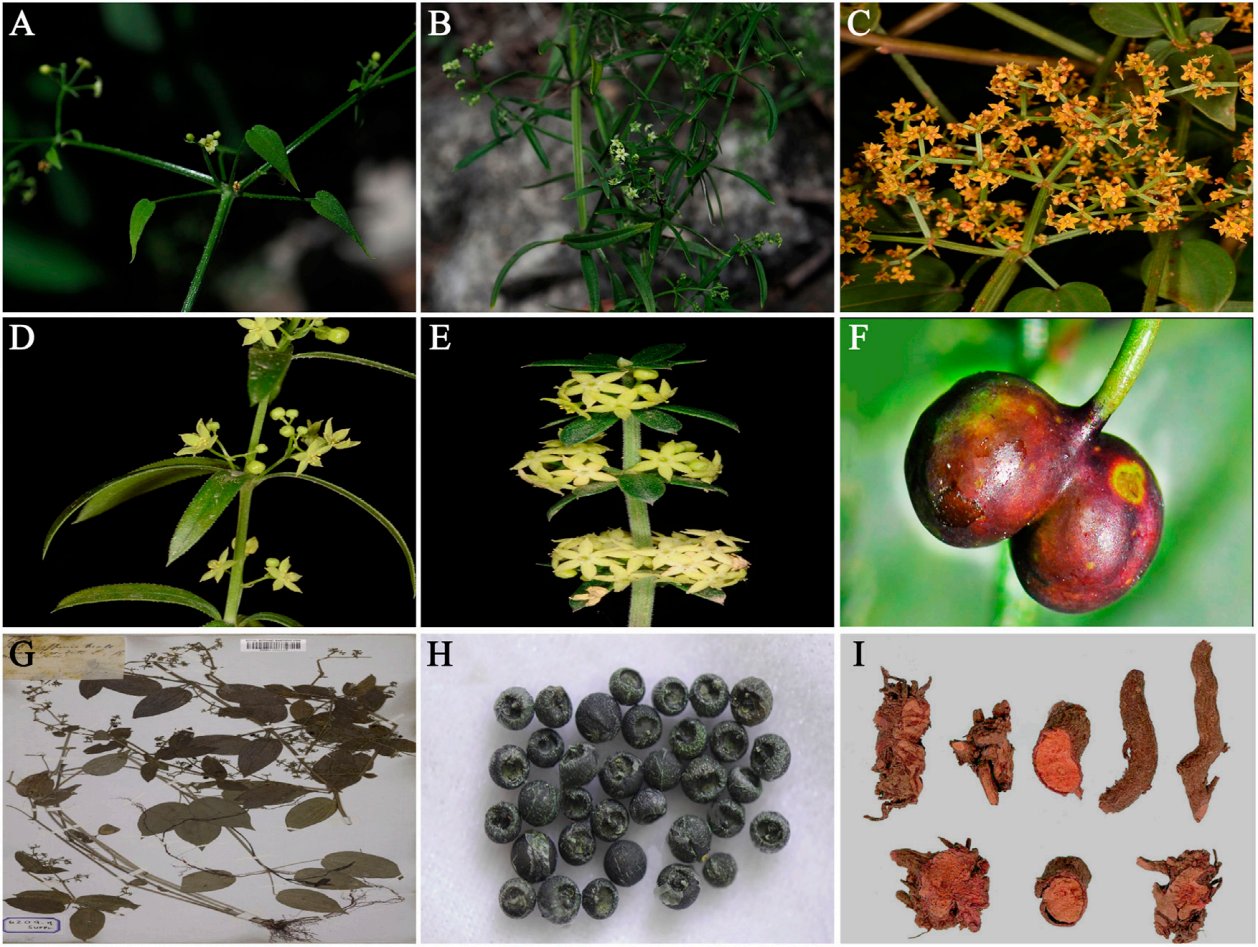
Botanical diagram of *R.* ***cordifolia*** (The images **(A,B,C,D,G)** are obtained from *Kew Botanical Garden* (http://powo.science.kew.org/):and **(F,H,I)** the *iPlant* (*ppbc.iplant.cn*). The whole plant of *R. cordifolia* **(A,B,C)**; the flowers of *R. cordifo*lia **(D,E)** the fruits of *R. cordifolia* **(F)**; The specimen of *R. cordifolia* **(G)**; dry fruit of *R. cordifolia* **(H)**; the dried root of *R. cordifolia* **(I)**.

The leaves of *R. cordifolia* are ovate or ovate-lanceolate, 2–6 cm long and 1–3 cm wide. Leaves in whorls of 4–8 or sometimes paired and blades lanceolate to broadly ovate, 0.7–8.5 cm long, 0.2–4.2 cm wide, acuminate at the apex, and rounded to cordate at the base, margins often with curved prickles, with recurved prickles and often pubescent as well. The plant morphology of *R. cordifolia* was shown in Figure 3. The flowers of *R. cordifolia* are characterized by cymes, located in the axilla or at the top, and are usually large pine cones in shape. Flowers glabrous, inflorescences usually numerous, scattered along stem, very loose to fairly dense, 0.5–2.5 cm long, pedicel 1–2.5 cm long, pedicel 0.2–6 mm long, bracts elliptic, 1.2–1.5 mm long, wide 0.3–0.4 mm. The calyx tube is 0.5–0.8 mm long and 0.8–1.4 mm wide. Corolla yellowish, green, with green cream or chartreuse, pink, or with purplish tips in buds, 4–6 mm wide, tube 0.2–0.8 mm long, lobes usually triangular, 1.5–3 mm long, wide 0.6–1.3 mm, apiculate, margin minutely papillary. Fruit glabrous, brownish black, lobes globose, 2.5–5 mm in diameter, pyrenes globose, 3 mm in diameter (Xun, 2014). The harvest season is generally in the third or fourth year of spring or autumn.

## Phytochemistry

Due to the extensive use of *R. cordifolia* in TCM, the chemical components and pharmacological effects of *R. cordifolia* have largely attracted the attention of scholars inside and outside the country. At present, hundreds of components have been isolated and identified from *R. cordifolia*. The reported chemical compounds of *R. cordifolia* were shown in Supplementary Table S1.

### Anthraquinones

Anthraquinones are a class of representative compounds in *R. cordifolia*, including alizarin, munjistin, rubiadin, purpurin, techoquinone and xanthopurpurin (King, 1992). At present, 28 anthraquinones were isolated from *R. cordifolia*. Dosseh et al. isolated four new anthraquinones from *R. cordifolia* root, which were 1-hydroxy 2-methoxy anthraquinone, 1,4-dihydroxy 2-methyl 5-methoxy anthraquinone or 1,4-dihydroxy 2-methyl 8-methoxy anthraquinone, 1,3-dimethoxy 2-carboxy anthraquinone and rubiadin (Dosseh et al., 1981a). Wang et al. used ethanol to extract *R. cordifolia* root and isolated seven anthraquinone compounds (2-methyl-1,3,6-trihydroxy-9,10-anthraquinone, 1-hydroxy-9,10-anthraquinone, 1,2,4-trihydroxy-9,10-anthrequinone, 2-methyl-1,3,6-trihydroxy-9,10-anthraquinone-3-*O*-*β*-D-glucoside, 1,2-dijhydroxy-9,10-anthraquinone-2-*O*-β-D-xylosyl (1→6)-*β*-D-glucoside and 1,3-dihydroxy-2-hydroxymethyl1-9,10-anthraquinone-3-*O*-*β*-D-xylosyl (1→6)-*β*-D-glucoside) (Wang et al., 1992). Above seventh compounds are a new class of compounds discovered. Their structures were elucidated to be 2-methyl-1,3,6-trihydroxy-9,10-anthraquinone-3-*O*-*β*-D-xylosyl (1→2)-*β*-D-(6′-*O*-acetyl) glucoside (Wang et al., 1992).

### Bicyclic hexapeptides

Bicycle hexapeptides are commonly considered as the most important bioactive compounds in *R. cordifolia*, which are extremely abundant. So far, 19 bicyclic peptides were found in *R. cordifoli*a. Lots of rubiaakane series peptides (RAs) compounds with anti-tumor bicyclic hexapeptides were isolated from *R. cordifolia*, such as RA-XVIII, RA-XII (Lee et al., 2008a). Up to now, numerous cyclic hexapeptides have been discovered and isolated from *R. cordifolia*. Each RA compound contains an 18-membered ring and a 14-membered ring system, which includes amino acids such as n-methyl-*O*-methyl-l-tyrosine, pyroglutamic acid, l-alanine and d-alanine. Among them, RA-V and RA-VII are the most abundant ingredients with 100 μg/g in *R. cordifoli*a, while the rest are less than 1 μg/g (Hitotsuyanagi et al., 2004). In addition, some RAs analogues or precursors have been isolated from *R. cordifolia* in recent years, such as neo-RA-V, allo-RA-V, *O*-seco-RA-V and *O*-seco-RA-XXIV (Hitotsuyanagi et al., 2012).

### Naphthoquinones

Naphthoquinone is a representative classification of components in *R. cordifolia*. To date, 16 naphthoquinones have been identified in *R. cordifolia*, such as mollugin (Wang et al., 2017), furomollugin (Ho et al., 1996), 2′-hydroxymollugin, 2′-methyoxymollogin, 1′,2′-dihydroxydihydromollugin, 1-methoxy-2-hydroxydihydromollugin (Hideji et al., 1993), epoxymollugin (Son et al., 2008), dihydromollugin and 2-carbomethoxy-2,3-epoxy-3-prenyl-1,4-naphthoquinone (CMEP-NQ) (Jun et al., 2011).

## Pharmacological activities

A number of researches have reported that *R. cordifolia* has numerous pharmacological activities, including anti-inflammatory, anti-cancer, anti-tumor, antioxidation, antibacterial, anti-platelet aggregation, anti-nephrotoxicity, anti-urolithiasis, hepatoprotective effects, and neuroprotective effects (Rawal et al., 2004; Joy and Nair, 2008; Divakar et al., 2010; Do et al., 2013; Gong et al., 2017; Luo et al., 2022). We summarized the pharmacological effects of *R. cordifolia in vitro* and *in vivo*, which were shown in Table 2 and Table 3.

**TABLE 2:** *In vitro* study on the pharmacological effects of *R. cordifolia.*

Bioactivities	Cell line	Compound/extract	Tested concentration	Active concentration	Positive control	Negative control	Result/mechanism	References
Antiadipogenic activity	3T3-L1 preadipocytes	2-Carbomethoxy-2,3-epoxy-3-prenyl-1,4-naphthoquinone (CMEP-NQ)	0, 10, 20, 40 μM	10, 20, 40 μM	Not available	Dulbecco’s modified eagle’s medium (DMEM)	CMEP-NQ (20, 40 μM) reduced viability of 3T3-L1 preadipocytes and mature adipocytes in a time- and dose-dependent manner. CMEP-NQ (10 μM) had no effect on the viability of these 2 cells, but the accumulation of less differentiation-related intracellular lipids was about 48.5%	(Jun et al., 2011)
							CMEP-NQ (10 μM) suppressed adipocytic differentiation of 3T3-L1 preadipocytes, and down-regulated the expression of transcription factors, including C/EBPα, PPARγ1 and PPARγ2	
Anti-cancer	HEp-2 cell	Methanol extract	5, 10.15, 20, 25, 30 mg/ml	5, 10.15, 20, 25, 30 mg/ml	Not available	DMEM	The viable cell rates of *R. cordifolia* methanol extract (5, 10, 15, 20, 25, and 30 mg/ml) for HEp-2 were 62.87, 54.67, 38.99, 26.92, 20.32, and 15%, respectively, and the control group was 79.06%. The methanol extract of *R. cordifolia* (5, 10, 15, 20, 25 and 30 mg/ml) with LD_50_ = 10 mg/ml inhibited the proliferation, promoted LDH release, reduced the levels of reduced GSH and GST, increased lipid peroxidation on human laryngeal carcinoma HEp-2 cells in a dose- dependent manner	Shilpa et al. (2012a)
	HER2-overexpressing SK-BR-3, BT-474 human breast cancer cells, SK-OV-3 human ovarian cancer cells	Mollugin	1, 5, 25, 50,100 μM	1, 5, 25, 50,100 μM	Not available	Dimethyl sulfoxide (DMSO)	Mollugin (1, 5, 25, 50 and 100 μM) inhibited HER2-overexpressing cancer cells with IC_50_ of 50 μM in a dose- and time-dependent manner. Inhibited cell proliferation and promoted apoptosis of breast and ovarian cancer cells by suppressing FAS expression through modulation of a HER2/Akt/SREBP-1c signaling pathway. Inhibited HER2 expression by suppression of NF-kB activation	Do et al. (2013)
Anti-inflammatory	RAW 264.7 cell	Mollugin	7.5, 15, 30 μM	7.5, 15, 30 μM	Not available	DMEM	In LPS-induced RAW264.7 inflammatory cells, mollugin (7.5, 15 and 30 μM) inhibited NO release, suppressed the expression of iNOS, IL-1β and IL-6 in a dose-dependent manner, and dose-dependent inhibition of LPS-induced activation of JAK2, STAT1 and STAT3 in RAW264.7 macrophages. Mollugin might be a JAK2 inhibitor that inhibited LPS-induced inflammatory response by blocking the activation of the JAK-STAT pathway	Zhu et al. (2013)
	LPS/IFN-g stimulated murine peritoneal macrophages	Methanol extract (1-hydrotectoquinone)	10, 20, and 40 μM	10, 20, and 40 μM	N^G^-monomethyl-L-arginine (L-NMMA)	RPMI-1640	The lowest tested concentration (10 μM) of 1-hydroxytectoquin could significantly inhibit the production of nitric oxide (NO•) compared to the control group treated with LPS/IFN-g. 1-Hydroxytectoquin (10, 20, and 40 μM) dose-dependently inhibited NO• production and iNOS expression in LPS/IFN-g stimulated murine peritoneal macrophages. These results were similar to the positive control group	Ghosh et al. (2010)
Antitumor	A375 cell, Hep2 cell, U937 cell, murine carcinoma	1-Hydroxytectoquinone (methanol extract)	10, 20, 40 μM	10, 20, 40 μM	Doxorubicin, camptothecin	RPMI-1640	1-Hydroxytectoquinone inhibited 50% of murine carcinoma (EAC) cell proliferation at less than 10 μM concentration, and inhibited the proliferation of A375 malignant skin melanoma cells with IC_50_ value of 3.2 μM. But relatively low toxicity against Hep2 cells (IC_50_ > 50 μM). The inhibitory effect on the U937 cell line was moderately cytotoxic with IC_50_ values of 19–28 μM	Ghosh et al. (2010)
Neuroprotective effects	mouse hippocampal HT22 cell	Mollugin	2.5, 5, 10 and 20 μM	2.5, 5, 10 and 20 μM	Trolox (50 μM)	DMEM	Mollugin (2.5, 5, 10 and 20 μM) promoted reactive oxygen species scavenging activity against glutamate-induced reactive oxygen generation in HT22 cells in a dose-dependent manner	Jeong et al. (2011)
							Mollugin (2.5, 5, 10 and 20 μM) suppressed pro-inflammatory mediators, including pro-inflammatory enzymes (iNOS and COX-2) and cytokines (TNF-α and IL-1β) in BV2 cells stimulated with LPS in a concentration-dependent manner	
							The neuroprotective effects of mollugin might be related to inhibition of pro-inflammatory mediators, upregulation of HO-1 expression and HO activity, nuclear accumulation of Nrf2, and activation of MAPK pathway	

**TABLE 3:** *In vivo* study on the pharmacological effects of *R. cordifolia*.

Bioactivities	Animal/model		Compound/Extract	Tested concentration	Effective concentration	Positive/Negative control	Result/Mechanism	Reference
Anti-inflammatory	Wistar rat/indomethacin-induced enterocolitis			300 mg/kg, 600 mg/kg body weight	300 mg/kg, 600 mg/kg body weight	Water	Reduced serum lactate dehydrogenase (LDH) activity levels	Pawar et al. (2011)
			Hydroalcoholic root extract					
	#212121; Wistar rat/trinitrobenzenesulfonic acid (TNBS)-induced colonic inflammation		Aqueous extract of the aerial part	250, 500, 1,000 mg/kg body weight	500 mg/kg body weight	Dexamethasone (0.3 mg/kg body weight)	Decreased the macroscopic damage area	Gong et al. (2017)
							Improved microstructure and reduced malondialdehyde content in the colon	
							Reduced levels of interleukin-1β (IL-1β) and tumor necrosis factor (TNF-α)	
	C57BL/6 mice/dextran sulfate sodium (DSS)-induced ulcerative colitis (UC)		Mollugin	10, 20, 40 mg/kg body weight	20, 40 mg/kg body weight	Distilled water	Reduced weight loss and the diseased activity index, ameliorated colon injury in ulcerative colitis (UC) mice	Li et al. (2020)
							Mollugin treatment (20 or 40 mg/kg) inhibited the production of pro-inflammatory cytokines IL-1β and TNF-α, and decreased the expression of IFN-γ and TLR4 in the DSS-induced UC mouse model	
Antioxidation	Wistar rat/aspirin plus pyloric ligation -induced ulcer		Chloroform extract/methanol extract	Methanol extract (100, 200, 400 mg/kg body weight)	400 mg/kg body weight		Reduced the ulcer index, total acidity, protein, and pepsin content of gastric juice, and increased mucoprotein content	Deoda et al. (2011)
				Chloroform extract (50,100,200 mg/kg body weight)		Ranitidine (10 mg/kg body weight)	Reduced lipid peroxidase (LPO) content, increased catalase (CAT), superoxide dismutase (SOD) and glutathione (GSH) content	
	Swiss albino mice		Ethanol extract	50,100 mg/kg body weight	50, 100 mg/kg body weight	Ethanol extract (100 mg/kg body weight)	Enhanced superoxide dismutase (SOD) and catalase (CAT) activities, increased glutathione (GSH) content, inhibited lipid peroxidase (LPO), reduced macrophage yield, macrophage viability, phagocytic index, serum immunoglobulin levels and renal PFC.	Lodi et al. (2011)
	Wistar rat/n-nitrosodiethylamine-induced experimental hepatocellular carcinogenesis	Methanol extract		250, 500, 750 mg/kg body weight	750 mg/kg body weight	Methanol extract (500 mg/kg body weight)	Reduced serum marker enzyme levels, including aspartate aminotransferase (AST), alanine aminotransferase (ALT), alkaline phosphatase (ALP), and lactate dehydrogenase (LDH).Decreased the level of LPO and hydroxyl radicals in liver	Shilpa et al. (2012b)
							Increased activity of several antioxidants in the liver, including SOD, CAT, glutathione peroxidase (GPx), glutathione S-transferase (GST), increased mitochondrial enzymes such as isocitrate dehydrogenase (ICDH), the level of succinate dehydrogenase	
Anti-tumor	Swiss albino mice/C57BL/6 mice	RC-18		1.25–5 mg/kg body weight	5 mg/kg body weight	Saline	Inhibited activity and proliferation of P388 cells and L1210 cells, but failed to show any inhibitory effect on solid tumors, Lewis lung cancer and sarcoma 180	Adwankar and Chitnis, (1982)
Anti-urolithiasis	Wistar albino rat/ethylene glycol induced urolithiasis	Hydro-alcoholic extract of root		286, 667 mg/kg body weight	667 mg/kg body weight	Cistone (750 mg/kg body weight)	Decreased calcium, oxalate levels and number of calcium oxalate crystals deposits in kidney tissue	Divakar et al. (2010)
Anti-nephrotoxicity	Swiss albino mice/cisplatin- induced renal damage	Hydro-alcoholic extract		250, 500 mg/kg body weight	500 mg/kg body weight	#212121; Hydro-alcoholic extract (500 mg/kg body weight)	Decreased values of serum urea and creatinine	Joy and Nair, (2008)
							Increased GPx, SOD and CAT.	
Hepatoprotective effects	Sprague-dawley Rat/carbon tetrachloride-induced liver injury	Rubiadin		50, 100, 200 mg/kg body weight	100 and 200 mg/kg body weight	Silymarin (100 mg/kg body weight)	Prevented the increase in malondialdehyde content and the decrease in reduced glutathione content in the liver of CCl_4_ poisoned rats in a dose-dependent manner	#080000; Rao et al. (2006)
							Histopathological examination confirmed the effective protective effect of rubiadin on carbon tetrachloride-induced liver injury in rats	
Neuroprotective effects	Sprague-dawley Rat/reserpine- induced movement disorders	Methanol extract		100, 200, 300 mg/kg body weight	Methanol extract (300 mg/kg) combined with Vitamin E (10 mg/kg)	Methanol extract (300 mg/kg)	Inhibited cavitary chewing movements, tongue protrusion, and increased the ability of exercise	Patil and Kasture, (2012)
							Increased the levels of SOD, CAT, and GSH in the forebrain region of rats, inhibited LPO and the level of dopamine	

### Anti-inflammatory effects

**FIGURE 4:**
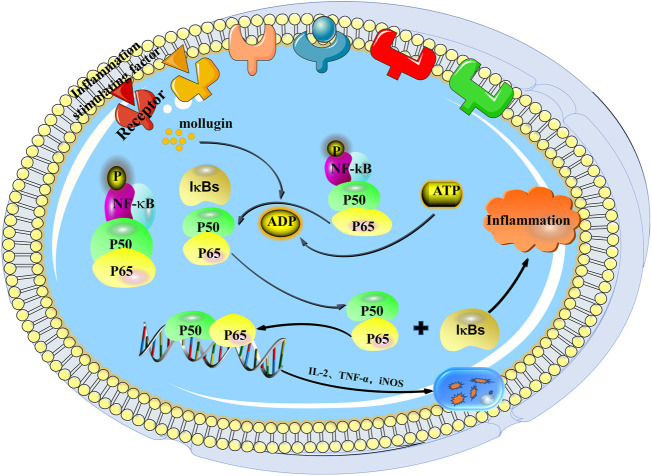
Anti-inflammatory mechanism of mollugin.

*R. cordifolia* has been used for the treatment of inflammatory diseases, such as colitis (Pawar et al., 2011), for a long time. In the study of indomethacin-induced enterocolitis in wistar rats, there were some acute intestinal inflammation effects, such as mesenteric haemorrhage, bowel wall thickening, mesenteric adhesion and multiple mucosal ulcerations of the small intestine, increased lactate dehydrogenase (LDH) activity, and so on. After treatment with hydro-alcoholic root extract of *R. cordifolia* (300 mg/kg and 600 mg/kg body weight) for 11 consecutive days, reduced intensity of lesions and inflammatory reaction in both ileum and colon tissue were observed, and reduced LDH activity was detected in indomethacin-induced enterocolitis rat model. These data suggested that *R. cordifolia* had a protective effect on indomethacin-induced colitis in rats, which may be used to treat patients with inflammatory bowel diseases (Pawar et al., 2011). Although the therapeutic effects of *R. cordifolia* on enterocolitis were investigated in rat, but the detection indexes were very simple, the evidences were weak, lacking of positive group, and the mechanism of action had not been confirmed, which should be investigated later. As described earlier, *R. cordifolia* was used to manage diarrhea in Chinese folk. Gong et al. investigated the anti-diarrheal and anti-inflammatory effects of the aqueous extract of *R. cordifolia’*s aerial part (AERCAP). They firstly measured the acute toxicity by acute oral toxicity test. *Via* LD_50_ study, they found that orally administered with graded doses of AERCAP (1, 2, 4, or 8 g/kg body weight) did not cause any mortality or obvious toxicity in the observation period. Orally administered with a single or maximum dose (13.2 g/kg body weight) of AERCAP also did not cause any mortality or behavioral and physical changes during the observation period, indicating that AERCAP had no acute oral toxicity in Male Swiss albino mice. Then, in a senna leaf-induced diarrhea mice model, administration with AERCAP (500 mg/kg and 1,000 mg/kg body weight) significantly inhibited the onset of semi-solid feces and reduced the evacuation index (EI) when compared with the model mice. But the effects of AERCAP was slightly weaker than the standard anti-diarrheal drug loperamide (4 mg/kg body weight). Finally, the anti-inflammatory activity of AERCAP was evaluated by a trinitrobenzenesulfonic acid (TNBS)-induced colonic inflammation rat model. They found that oral treatment with AERCAP or positive drug dexamethasone (0.3 mg/kg body weight) decreased the macroscopic damage area, improved the microscopic structure, reduced the malondialdehyde (MDA) content and levels of pro-inflammatory cytokines tumor necrosis factor (TNF-α) and interleukin-1β (IL-1β) in colonic tissue when compared with the model rat (Gong et al., 2017). The study provided the scientific evidences of the traditional use of AERCAP for treating diarrhea in folk, but did not elucidate its underlying mechanisms. Besides, the dose of 500 and 1,000 mg/kg of AERCAP exhibited a great therapeutic effect against diarrhea, but the high dose of AERCAP (2,000 mg/kg body weight) had no effects on senna leaf-induced diarrhea in mice. This was a common phenomenon in crude extract preparation. Although the acute toxicity of AERCAP was detected by observation of mortality, behavioral and physical changes, the systemic toxicity should be measured through viscera indexes, H&E staining of primary organs, as well as biochemical indicators of peripheral blood. The systemic toxicity might be an influence factor on the therapeutic effects of AERCAP on diarrhea. Mollugin, a major compound of *R. cordifolia*, was reported to possess an anti-inflammatory activity (Figure 4). MTT assay was performed to assess the cytotoxicity of mollugin on RAW264.7 cells and showed that the different concentration of mollugin (7.5, 15 and 30 μM) had no cytotoxicity on RAW264.7 cells after treatment for 24 h. In lipopolysaccharide (LPS)-induced RAW264.7 inflammatory cells, mollugin (7.5, 15 and 30 μM) was able to inhibit nitric oxide (NO) release, suppress the expression of nitric oxide synthase (iNOS), IL-1β and IL-6 in a concentration-dependent manner. Mechanically, mollugin (7.5, 15 and 30 μM) dose-dependently inhibited the activation of JAK2, STAT1 and STAT3 induced by LPS in RAW264.7 macrophages. Molecular docking analysis further demonstrated that mollugin exhibited a high binding ability with JAK2, and the binding pattern was similar to AG490, a specific JAK2 inhibitor, indicating that mollugin was a JAK2 inhibitor. These data suggested that mollugin inhibited LPS-induced inflammatory responses in RAW264.7 macrophages *via* blocking the activation of the JAK-STAT pathway (Zhu et al., 2013). This finding is interesting because they demonstrate the direct target of mollugin against inflammatory response. However, the binding between mollugin with JAK2 should be proved by experiments, such as surface plasmon resonance technology (SPR), cellular thermal shift assay (CETSA) (Martinez et al., 2013) or isothermal dose-response fingerprint (ITDRF_CETSA_) (Chang et al., 2016), and the *in vivo* anti-inflammatory activity of mollugin should be investigate. Moreover, the authors confirms the anti-inflammatory activity of mollugin, but the lack of positive control reduces reliability of research which requires further validation. Another study demonstrated that mollugin could also ameliorated dextran sulfate sodium (DSS)-induced ulcerative colitis (UC) in C57BL/6 mice. Intragastric administrated with mollugin (10, 20, and 40 mg/kg body weight) significantly reduced weight loss and the diseased activity index, ameliorated colon injury in UC mice. In addition, mollugin treatment (20 or 40 mg/kg body weight) markedly inhibited the production of pro-inflammatory cytokines IL-1β and TNF-α, and decreased the expression of interferon γ (IFN-γ) and toll-like receptor (TLR4) in the DSS-induced UC mouse model. Therefore, the improvement of DSS-induced ulcerative colitis by mollugin might be by suppressing the production of pro-inflammatory cytokines (Li et al., 2020). 1-hydrotectoquinone (10, 20 and 40 μM) was reported to reduce nitric oxide (NO•) production in LPS and IFN-g stimulated murine peritoneal macrophages, and inhibit iNOS expression in LPS induced cultured murine peritoneal macrophages in a dose dependent manner (Ghosh et al., 2010). The *in vivo* activity and mechanistic investigation of 1-hydrotectoquinone against inflammatory need for further study. Furthermore, in addition to the drug group and the negative control group, the positive group should be designed to enhance the reliability of the study and the rationality of the experiment. So far, there is a relatively wide selection of anti-inflammatory positive drugs, such as aspirin, celecoxib, diclofenac, diflunisal and ibuprofen etc (Khandia and Munjal, 2020). Taken together, these findings demonstrate that *R. cordifolia* extract and part of its compounds have anti-inflammatory activity in different models, but the mechanism of regulating inflammatory was still unclear. Further research and clinical trial data are needed to confirm its anti-inflammatory activity and mechanism of action.

### Anti-cancer effects

Although great progress has been made in various anti-cancer treatments, such as targeted therapy and immunotherapy (Torre et al., 2016), chemotherapy is still the most commonly used treatment (Bukowski et al., 2020). The methanol extract of *R. cordifolia* (5, 10, 15, 20, 25 and 30 mg/ml) with LD_50_ = 10 mg/ml could inhibit the proliferation, promote LDH release, reduce the levels of reduced glutathione (GSH) and glutathione transferase (GST), increase lipid peroxidation on human laryngeal carcinoma HEp-2 cells in a dose dependent manner. Moreover, *R. cordifolia* extract (30 mg/ml) could induce apoptotic cell death of HEp-2 cells, indicating its potential for treating laryngeal squamous cell carcinoma (Shilpa et al., 2012a). The study of *R. cordifolia* against laryngeal squamous cell carcinoma just using 1 cell line is not sufficient, the other cell lines of laryngeal squamous cell carcinoma and xenograft mouse model should be used to assess the activity of *R. cordifolia*. Moreover, this study lacks a positive control, and this result needs to be further validated. A cyclic hexapeptide compound (RC-18) (1.25, 2.5 and 5 mg/kg body weight) isolated from *R. cordifolia* had been demonstrated to had a significant anti-cancer effect against ascites tumors L1210, P338 and L5178Y, as well as solid tumor B16 melanoma, which could prolong median survival time in corresponding tumor mice models. RC-18 had no inhibitory effect on two solids tumors, Lewis lung carcinoma and sarcoma 180, which had no remarkable effect on mean tumor weight (Adwankar and Chitnis, 1982). This study reveals the ability of RC-18 against a spectrum of murine tumor models. A number of natural products have activities against cancer or tumor in mice model, but have no therapeutic actions on humans. P388 and L1210 tumor mice models used in this study have got predictive value in the clinic. Plenty of compounds are able to inhibit P388 and L1210, which are later demonstrated to have activity against cancer or tumor in clinic, indicating RC-18 may be a promising agent against cancer for the clinical use. The toxicity study, mechanism research and clinical research of RC-18 should be carried out in the further. Mollugin had been shown preclinical anti-cancer actions in varieties of cancer models. Mollugin (20, 40, 60 and 80 μM) was able to suppress cell viability of U251MG and U87MG cells (glioblastoma cells), MKN45 cells (gastric cancer cell), MCF-7 cells (breast cancer cell), A549 cells (lung cancer cell) and HT29 cells (colon cancer cell) in a concentration- and time-dependent manner, but had no effect on cell viability of mouse primary neurons. Mollugin (10, 20, and 40 μM) could induce mitochondria apoptosis and autophagy in glioblastoma cells by inhibiting PI3K/AKT/mTOR/p70S6K and ERK signaling pathways (Zhang et al., 2014). The experimental design of this study is rigorous. The multiple cancer cell lines and mouse primary cell were used to evaluate the cell viability of mollugin on cancer cells and normal cells. For mechanism research, the pan-caspase inhibitor Z-VAD, RNA interference with shRNA for knockdown an autophagic regulator Beclin-1, PI3K kinase inhibitor LY294002, and ERK inhibitor U0126 were carried out to clarify the relationship between mollugin with apoptosis and autophagy, as well as the mechanism of action of mollugin. These evidences are solid. However, there are still many questions need to be solve. For instance, what’s the relationship between apoptosis and autophagy induced by mollugin in glioblastoma cells. There may be a switch locates in the upstream of PI3K/AKT/mTOR/p70S6K and ERK signaling pathways, which is regulated by mollugin that should be identified. Moreover, the *in vivo* study is extremely necessary to carry out to prove the anti-cancer ability of mollugin. Mollugin (1, 5, 25, 50, and 100 μM) was also reported to possess the anti-cancer abilities in HER2-overexpressing cancer cells in a dose- and time-dependent manner with an IC_50_ value of 50 mM. Further study demonstrated that mollugin inhibited cell proliferation and promoted apoptosis of breast and ovarian cancer cells by suppressing FAS expression through modulation of a HER2/Akt/SREBP-1c signaling pathway. In addition, mollugin could inhibit HER2 expression by suppression of NF-kB activation (Do et al., 2013). In summary, these findings suggest that *R. cordifolia* and its ingredients may be clinically useful as anti-cancer agent. The *in vivo* activities against cancers should be explored using cancer animal models. And authors ought to design a positive drug group in the study. For example, paclitaxel is known as one of the most successful natural anticancer drugs (Zhu and Chen, 2019). Vinblastine, podophyllotoxin and camptothecin have also been used as anticancer drugs in clinical practice (Gezici and Sekeroglu, 2019), which is conducive to enhance the credibility, scientificity and rationality of the article.

### Anti-tumor effects

Many studies had shown that cyclic peptides were bioactive compounds responsible for anti-tumor activity of *R. cordifolia*. The majority of the RA series compounds indicated cytotoxicity against a number of tumor cells, including P-388 leukaemia cells, SGC-7901 human gastric adenocarcinoma cells, A-549 human non-small cell lung carcinoma cells, and HeLa (human cervical carcinoma) cells (Chen et al., 2015). For example, RA-XVIII (IC_50_ = 0.012 μg/ml) (Lee et al., 2008a), RA-XXIII (IC_50_ = 0.16 μg/ml) and RA-XXIV (IC_50_ = 0.48 μg/ml) (Lee et al., 2008b) could effectively inhibit the proliferation of P-388 leukaemia cells. The studies of the RA series compounds are pretty simple, which all focus on their cytotoxicity on several tumor cell lines. Whether they have significant cytotoxicity on normal cells should be evaluated. The *in vivo* activities of RA series compounds against above tumors are the core for further study. Wang et al. found that mollugin isolated from *R. cordifolia* root had anti-tumor properties. Mollugin (20, 40, and 80 μM) could not only significantly inhibit the expression of NF-κB receptors induced by TNF-α in a dose-dependent manner, but also inhibit p65 phosphorylation and nuclear metastasis induced by TNF-α, phosphorylation and degradation of κB inhibitor (IκBα), and IκB kinase (IKK) phosphorylation. Moreover, mollugin (20, 40, and 80 μM) could inhibit the proliferation of HeLa cells. *Ex vivo* experimental showed that mollugin (25 or 75 mg/kg body weight) effectively inhibited the growth of heterogeneous transplanted tumors from HeLa cells. The author concludes that mollugin might be a potential drug for treating cancer by targeting NF-κB (Wang et al., 2017). There are some questions need to be solved. For example, what’s the direct target of mollugin against cancer? Both TNFR1 and NF-κB are potential targets of mollugin. The inhibitors of TNFR1 and NF-κB can be used to demonstrate whether mollugin have an anti-tumor effect through regulation of TNFR1 or NF-κB. Besides, the direct interaction relationship between mollugin with TNFR1 or NF-κB should be verified by molecular docking simulation, molecular dynamics simulation, SPR, CETSA or ITDRF_CETSA_. 1-hydroxytectoquinone was another active ingredient in *R. cordifolia*, which could inhibit 50% of murine carcinoma (EAC) cell proliferation at less than 10 μM concentration. In addition, it had an inhibitory effect on A375 malignant skin melanoma cells with an IC_50_ value of 3.2 μM (Ghosh et al., 2010). The study of 1-hydroxytectoquinone only on several tumor cell lines are not enough. Also, this study lacks positive control. Epothilone, a newly developed antitumor drug, can be used as a positive drug in antitumor experiments (Cheng and Huang, 2018). The widely research of 1-hydroxytectoquinone against tumor on xenograft mice model should be initiated. Overall, the underling molecular mechanisms of the ingredients of *R. cordifolia* against tumors need to be further studied in the future.

### Antioxidation effects

**FIGURE 5:**
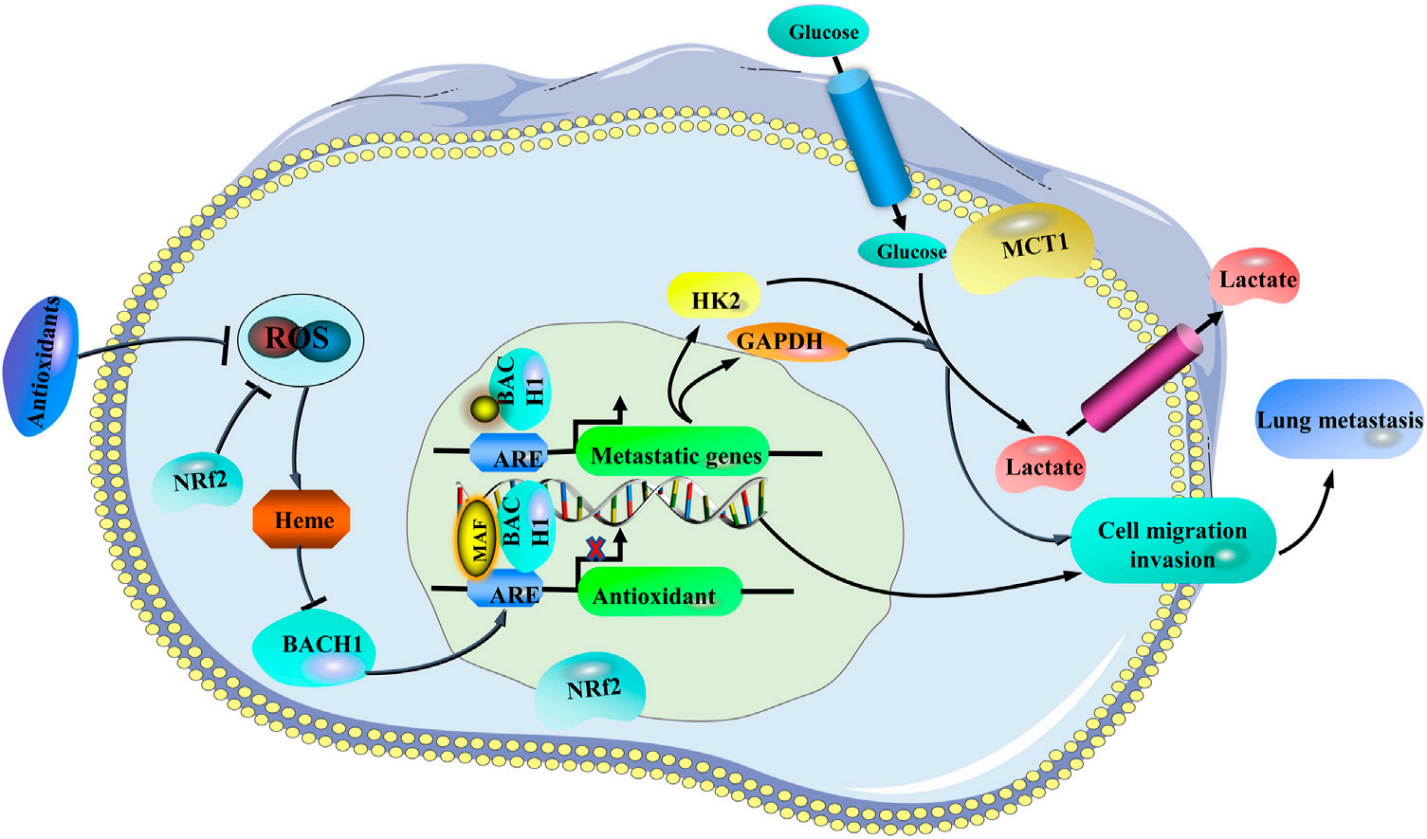
Antioxidative mechanisms.

Natural antioxidants are a variety of potential plant drug resources, with a long history of ethnopharmacology (Dkhil et al., 2016). Therefore, it is vital to obtain antioxidant components from natural medicines. Study had demonstrated that *R. cordifolia* had antioxidant activity, the hydroxyl group on the benzene ring in *R. cordifolia* played a crucial role in scavenging free radicals, while the hydroxyl structure in hydroxyanthraquinone could effectively enhance the free radical scavenging ability (Cai et al., 2004). The antioxidant mechanism (Lignitto et al., 2019) was shown in Figure 5. Deoda et al. found that methanolic extract of *R. cordifolia* (100, 200 or 400 mg/kg body weight) and chloroform fraction of *R. cordifolia* (50, 100 or 200 mg/kg body weight) brought notable decrease of ulcer index, total acidity, protein, pepsin content of the gastric fluid, and increased of the mucin content in an aspirin plus pylorus-ligated ulcer rat model. Several key antioxidant parameters, lipid peroxidase (LPO) content was reduced, catalase (CAT), superoxide dismutase (SOD) and GSH contents were elevated by methanolic extract of *R. cordifolia* (100, 200 or 400 mg/kg body weight) and chloroform fraction of *R. cordifolia* (50, 100 or 200 mg/kg body weight) treatment. In above study, the standard antiulcer drug ranitidine (10 mg/kg body weight) was used as positive drug. It was concluded that the gastroprotective effect of *R. cordifolia* was partly due to its antioxidant activity (Deoda et al., 2011). Lodi et al. assessed the antioxidant activity of the ethanolic extract of the roots of *R. cordifolia in vivo*. Lead nitrate (40 mg/kg body weight) treatment cause an increase in LPO, CAT and GSH contents, reduction in macrophage yield, viability of macrophage, phagocyte index, serum immunoglobulin level, and PFC in kidney in Swiss albino mice. However, the ethanolic extract of the roots of *R. cordifolia* (50 and 100 mg/kg body weight) administration significantly reversed lead nitrate-induced toxicity on oxidative stress and immunological parameters, demonstrating that *R. cordifolia* had an antioxidant property (Lodi et al., 2011). Shilpa et al. reported that the methanol extract of *R. cordifolia* (750 mg/kg body weight) could reduce levels of serum marker enzymes including aspartate transaminase (AST), alanine aminotransferase (ALT), alkaline phosphatase (ALP) and LDH, decrease the level of LPO and hydroxyl radicals in liver, increase the activities of several antioxidants including SOD, CAT, glutathione peroxidase (GPx), GST in liver, elevate the levels of mitochondrial enzymes like isocitrate dehydrogenase (ICDH), succinate dehydrogenase (SDH), α-ketoglutarate dehydrogenase (α-KGDH) in a N-nitrosodiethylamine-induced experimental hepatocellular carcinogenesis rat model. These data demonstrated that *R. cordifolia* might be applied as an antioxidant for the treatment of some cancers (Shilpa et al., 2012b). In above studies, the antioxidant activity of *R. cordifolia* are demonstrated by detection of several antioxidant indexes, however, the reason why *R. cordifolia* possesses the antioxidant ability should be further study. In addition, it is necessary to include a positive drug such as vitamin E in the experiment to make the results more convincing (Mohd et al., 2020). Besides, what are the antioxidant active components of *R. cordifolia* need to be confirmed. Overall, these studies demonstrate the antioxidation effects of *R. cordifolia* or its ingredients in different models, but none of them carry out the mechanism research, which require further study.

### Antibacterial effects

Antibacterial drugs are an important class of therapeutic drugs used to treat bacterial infectious diseases (Sun et al., 2004). Unnecessary and overuse of antibiotics is of particular concern, as this could lead to several adverse drug events, including end-organ toxicity, allergic reactions, subsequent infection with drug-resistant organisms, and *Clostridium difficile* infection (Tamma et al., 2017). The demand for new antibacterial drugs that are effective against drug-resistant microorganisms has greatly increased (Wright et al., 2017). The plant materials are generally preferred now for use as natural antibacterial agents in the treatment of various infections (Ginovyan et al., 2020). Basu et al. found that the methanol extracts of *R. cordifolia* (1, 2.5, 5, 7.5, and 10 mg/ml) exhibited activity against 6 g-positive bacteria (*Bacillus cereus*, *Bacillus pumilus*, *Bacillus subtilis*, *Micrococcus luteus*, *Mycobacterium luteum* and *Mycobacterium luteum*) in a concentration-dependent manner. Besides, the methanol extracts of *R. cordifolia* (1, 2.5, 5, 7.5, and 10 mg/ml) could also inhibit *Pseudomonas aeruginosa* in a concentration-dependent manner. Mover, the water extracts of *R. cordifolia* (1, 2.5, 5, 7.5, and 10 mg/ml) could only inhibit *Bacillus subtilis* and *Staphylococcus aureus*. All these bacterial were highly susceptible to inhibition by streptomycin (0.1 mg/ml) used as standards, and part of them were significantly inhibited by penicillin G (0.01 mg/ml) used as positive drug (Basu et al., 2005). The study demonstrates the excellent antibacterial ability of *R. cordifolia* and shows the methanol, methanol and water extracts of *R. cordifolia* can inhibit different bacteria. The reason why different extracts of *R. cordifolia* exhibit different antibacterial abilities should be explained. The comprehensive chemical profiling of *R. cordifolia* is worthy of being explored by using chemical analysis method, such as two-dimensional mixed-mode liquid chromatography × reversed-phase liquid chromatography system (Dai et al., 2022), which is beneficial to antibiotic discovery from *R. cordifolia*.

### Anti-platelet aggregation effects

Thromboembolic disease is one of the most common clinical diseases (Nagalla and Bray, 2016). Platelet aggregation is an important cause of thrombosis. The research and exploration of anti-platelet aggregation drugs is still an important topic of concern (Xie et al., 2019). There are a few reports on anti-platelet aggregation effects of *R. cordifolia*. Research found that the partially purified fraction of *R. cordifolia* could inhibit rabbit platelet aggregation induced by platelet activating factor (PAF), and inhibit the binding of 3H-PAF to platelets (Tripathi et al., 1993). Although this study had demonstrated the anti-platelet aggregation effects of *R. cordifolia*, further pre-clinical research on the effect of *R. cordifolia* on thrombosis in mice, and clinical research should be performed to confirm their mechanism of action and therapeutic effects in clinic.

### Hepatoprotective effects

The liver is an important organ for the regulation of human metabolism. Hepatic damage is associated with disorder of metabolic functions (Limon et al., 2021). Liver ailments are still a worldwide health problem (Venkateswaran et al., 1997; Daniyal et al., 2019). At present, the use of herbal medicine to treat liver ailments has huge potential. Rao et al. evaluated the effect of rubiadin isolated from *R. cordifolia* on carbon tetrachloride (CCl4)-induced liver injury in rats. They found that rubiadin (50, 100, and 200 mg/kg body weight) significantly prevented the increase in malondialdehyde content and the decrease in reduced glutathione content in the liver of CCl_4_ poisoned rats in a dose-dependent manner. The hepatoprotective effects of rubiadin were similar to silymarin (100 mg/kg body weight), a known hepatoprotective compound. Histopathological examination of rat liver sections also confirmed that rubiadin had an effective protective effect on rat liver injury induced by carbon tetrachloride (Rao et al., 2006). Although rubiadin has a protective effect on liver damage, its mechanism of action is still unclear, which needs to investigate in further study. Except for rubiadin, other hepatoprotective compounds of *R. cordifolia* is necessary to identified.

### Neuroprotective effects

The neuroprotective effects of *R. cordifolia* have been studied. Patil et al. found that methanol extract of *R. cordifolia* (100, 200, and 300 mg/kg body weight) combined with vitamin E (10 mg/kg body weight) treatment significantly inhibited cavitary chewing movements, tongue protrusion, and increased the ability of exercise in a reserpine-induced orofacial dyskinesia model. The authors also found that *R. cordifolia* treatment could increase the levels of SOD, CAT, and GSH in the forebrain region of rats, inhibit LPO, and increase the level of dopamine. However, the dose alone methanol extract of *R. cordifolia* (300 mg/kg body weight) did not produce any significant change. These findings suggested that *R. cordifolia* had a neuroprotective effect (Patil and Kasture, 2012). It is worth noting that *R. cordifolia* and vitamin E co-treatment play a role in protecting animals against reserpine-induced orofacial dyskinesia. But *R. cordifolia* treatment alone don’t have any effects on the disease. It is known that vitamin E, a free-radical scavenger, can improve symptoms of antipsychotic-induced tardive dyskinesia (Xu et al., 2022). It is highly possible that *R. cordifolia* may strengthen the therapeutic effect of vitamin E on neuroleptic induced orofacial dyskinesia. Therefore, *R. cordifolia* may be an adjuvant therapy when combined with vitamin E for treating antipsychotic-induced tardive dyskinesia. Rawal et al. showed that *R. cordifolia* (2 mg/ml) exerted neuroprotective properties through elevating the GSH levels by promoting GCLC expression, directly scavenging free radicals, and inhibiting the expression of iNOS gene which was essential for neuronal injury during hypoxia/ischemia in rat hippocampal slices subjected to oxygen glucose deprivation (Rawal et al., 2004). Mollugin (2.5, 5, 10, and 20 μM) could also promote reactive oxygen species scavenging activity against glutamate-induced reactive oxygen generation in HT22 cells in a concentration-dependent manner. In this study, trolox (50 μM) was used as a positive drug, which showed an obvious cytoprotective effect and reactive oxygen species scavenging activity. Besides, mollugin (2.5, 5, 10, and 20 μM) was able to suppress pro-inflammatory mediators, including pro-inflammatory enzymes [inducible nitric oxide synthase (iNOS) and cyclooxygenase-2 (COX-2)] and cytokines (TNF-α and IL-1β) in BV2 cells stimulated with LPS in a concentration-dependent manner. Its neuroprotection might be mediated by the effects on inhibition of pro-inflammatory mediators, up-regulation of heme oxygenase-1 (HO-1) expression and the heme oxygenase (HO) activity, nuclear accumulation of Nrf2 and activation of p38 mitogen-activated protein kinase (MAPK) pathway (Jeong et al., 2011). The study only demonstrates the neuroprotective effect of mollugin *in vitro*, the more solid evidence should be acquired form animal model of neurological disease.

## Toxicity

Although a large number of research have proved that *R. cordifolia* has numerous pharmacological activities, some compounds isolated from this plant are still toxic. Furthermore, it is extremely necessary to investigate its toxicological effects in order to determine the clinical use of the drug. The toxicity of the crude ethanol extracts of *R. cordifolia* fruit (100, 500, and 1,000 mg/kg body weight) was evaluated by biochemical parameters and histopathological changes and found that it had harmful effects on liver. The LD_50_ value was higher than 1,000 mg/kg. In addition, dibutyl phthalate identified from *R. cordifolia* fruits was showed a holistic toxicity *via in silico* analysis (Anantharaman et al., 2016). However, this experimental time is short, and further in-depth studies and developmental toxicity studies are needed to determine the effects of *R. cordifolia* on long-term administration to animals. In addition, its toxic dose can’t be determined by animal toxicity studies, and clinical trials are needed to evaluate its safety in humans. Studies had found that rubiadin, a compound in *R. cordifolia*, had carcinogenic potential. A rat medium-term multi-organ carcinogenesis model was used and Male F344 ⁄ DuCrj rats were given 0.008% or 0.04% containing diet. The effects of rubiadin on the kidney, liver, and large intestine were explored. The results showed that rubiadin treatment increased renal cell adenomas and carcinomas in the outer medulla of the kidney, preneoplastic lesions, hepatocellular foci, dysplasia, as well as adenomas and adenocarcinomas in the large intestine (Inoue et al., 2009). Therefore, we should avoid exposure to carcinogenic substances containing rubiadin.

### Pharmacokinetics

Up to now, there are few reports on the pharmacokinetics of *R. cordifolia*. Gao et al. used Ultra High- Performance Liquid Chromatography-tandem Mass Spectrometry (UHPL-MS/MS) method to simultaneously determine munjistin, purpurin, and mollugin in rat plasma. The Sprague-Dawley (SD) rats (220–20 g body weight) were oral administrated with *R. cordifolia* (0.82 g/kg body weight). The 0.4 ml of blood were collected from the orbital venous plexus at the indicated time points (0, 0.33, 0.67, 1, 1.3, 1.67, 2, 2.5, 3, 4, 6, 8, 12, and 24 h) and the plasmas were collected for pharmacokinetic study. The results showed that after treatment with *R. cordifolia*, the maximum plasma concentrations (*C*_max_) for purpurin, munjistin and munjistin were 70.10–11.78 ng/ml, 26.09–6.6 ng/ml, and 52.10–6.71 ng/ml, respectively. The time for maximal concentration (*T*_max_) for purpurin, munjistin and mollugin were 1.61–0.24 h, 2.58–0.19 h, and 1.99–0.21 h, respectively (Gao et al., 2016). Except for this study, more pharmacokinetic studies should be performed in order to better understand the absorption, distribution, metabolism and excretion of the active ingredients in *R. cordifolia*.

## Clinical application

Hemorrhage syndrome is a common symptom in clinic, such as acute hemorrhage, cerebral hemorrhage, vascular rupture and bleeding (Dima et al., 2018). As a traditional Chinese medicine, *R. cordifolia* has been used for many years in the treatment of hemorrhage in clinical (Chen, 2013). *R. cordifolia* could effectively treat most hemorrhages, such as hematemesis, hematuria, dysmenorrhea, and abnormal uterine bleeding (Li et al., 2009). In a clinical research, 110 patients (25–49 years old) with abnormal uterine bleeding caused by intrauterine device were treated with *R. cordifolia* decoction (18 g/d) or control drug *yunnanbaiyao* (1.5 g/d). The blood routine examination and blood coagulation were evaluated of each patient before and after medication. They found that the total effective rate of *R. cordifolia*-treated group was 94.5%, which was higher than the *yunnanbaiyao*-treated group (81.8%). The hemostatic ability of *R. cordifolia*-treated group was much stronger than that of *yunnanbaiyao*-treated group. The platelet levels of two drug-treated groups were much higher than those before treatment. *Yunnanbaiyao* is a famous traditional Chinese medicine which is widely used in clinic for treating various bleeding disorders in China. The better performance of *R. cordifolia* in treating abnormal uterine bleeding compared with *Yunnanbaiyao* suggests that *R. cordifolia* has a huge potential for clinical application. The clinical research of *R. cordifolia* for the treatment of other bleeding disorders is worthy of investigation (Hu, 2019). This study is meaningful. The therapeutic schedule should be further optimized. For instance, the accepted drug for treating abnormal uterine bleeding in the domestic and overseas, such as tranexamic acid should be used as positive drug in patients (Bradley and Gueye, 2016). The treatment groups should be divided into low, medium, and high dose of *R. cordifolia*, which can demonstrate the optimal treatment dosage. Also, combination therapy of *R. cordifolia* and other effective drugs is worthy of in-depth study. In another clinical study, the decoction of *R. cordifolia* combined with other herbs were used to treat dysfunctional uterine bleeding. 184 patients (18–51 years old) participated in the research. Among them, 64 patients were treated with norethisterone, a synthetic hormone for treating a range of menstrual problems in clinic. 120 patients were treated with *R. cordifolia* decoction. They found that the total effective rate of in *R. cordifolia*-treated group was 90%, and the norethisterone-treated group was 78.1%. Therefore, according to this clinical data, it can prove that *R. cordifolia* combined with other drugs are able to effectively treat dysfunctional uterine bleeding (Table 4) (Zhang, 2011). Due to the hemostatic effect of *R. cordifolia* and the strongly wound healing effect of *Bletilla striata* Reichb. f, the two drugs were used in combination to treat vaginal bleeding and wound healing after hysterectomy (Li, 2011). However, the detailed data of these clinical studies were not exhibited in any scientific literatures. Although *R. cordifolia* has been used in clinical for many years, further research is needed on the medicinal value and clinical application of *R. cordifolia*, in order to discover its medicinal value potential and efficacy and improve the safety and use of medicine.

**TABLE 4:** Clinical application of *R. cordifolia*.

Function	Symptoms	The number of patients	Age	Dose		Effective rate	References
				*R. cordifolia*	Control drug		
Treatment of abnormal uterine bleeding	Uterine bleeding caused by intrauterine device	110 patients. (50 patients were treated with *R. cordifolia* and 50 patients were treated with *yunnanbaiyao*)	25–49 years old	*R. cordifolia* decoction. (18 g/d)	*Yunnanbaiyao.* (1.5 g/d)	*R. cordifolia*-treated group was 94.5%, and *yunnanbaiyao*-treated group was 81.8%	Hu, (2019)
Treatment of dysfunctional uterine bleeding	Dysfunctional uterine bleeding	184 patients. (120 patients were treated with *R. cordifolia* and 64 patients were treated with norethisterone)	18–51 years old	*R. cordifolia* decoction. (800 ml/d)	Norethisterone. (2.5–20 mg/d)	*R. cordifolia*-treated group was 90%, and norethisterone- treated group was 78.1%	Zhang, (2011)

## Perspectives and discussion

As a famous TCM, *R. cordifolia* has high commercial and medicinal value. *R. cordifolia* is one of the earliest and most widely used red dyestuff in ancient China. People also use *R. cordifolia* for treating a wide variety of diseases in folk for thousand years. Due to its excellent therapeutic effects and few side effects in clinic, the plant has received increasing attention in recent years. More than 100 compounds of *R. cordifolia* are identified. The pharmacological activities of *R. cordifolia* and its compounds were explored. All these findings are beneficial for drug discovery form *R. cordifolia* and lay the foundation for its clinical application. However, there are still a number of questions about *R. cordifolia* research need to be solved. The following aspects should be considered in future study.

Firstly, until now, *R. cordifolia* don’t has its own genetic identity card. The genomic information of *R. cordifolia* has not been uncovered. It is necessary to perform genomic sequencing and acquire genome sequence of *R. cordifolia*, which is essential for identification of key active ingredients, exploration of synthesis pathway of the active compounds, quality improvement, as well as its industrial production. Moreover, genomic sequencing should combine with comparative genomics, metabonomic and transcriptomics, which can give more information of *R. cordifolia* itself. All these explorations will give *R. cordifolia* a pair of wings to fly to modernization of TCM.

Secondly, although more than 100 compounds of *R. cordifolia* have been identified, pharmacologic actions of the most compounds have not been explored. For instance, *R. cordifolia* is traditionally applied for treating various hemorrhage syndromes, but its active compounds in stopping bleeding are largely unknown. Conversely, several compounds with anti-platelet aggregation effects are found. As is well known, pharmaceutical activity screening form TCM is usually a time-consuming, labor-intensive and cost-intensive process. Fortunately, the artificial intelligence (AI) brings a new dawn for medical field, which has excellently facilitated the modern drug discovery (Gupta et al., 2021). In our previous study, we developed a drug screening model based on machine learning (ML), a primary subfield of AI, to high-throughput screening the active compounds in promoting platelet production from thousands of natural compounds of TCM. As a result, we found indenol had a high activity to accelerate platelet production and exhibited an excellent hemostatic ability in radiation-induced thrombocytopenia mice (Wang L. et al., 2022). We believe that drug screening model based on AI algorithm is a powerful tool which can be applied to identify the active compounds from *R. cordifolia* in treating hemorrhage syndromes as well as other diseases. Except for AI high throughput virtual screening, other drug screening method can be used to *R. cordifolia* study, such as reporter gene system, high-throughput microscopy assays.

Thirdly, almost all the research on *R. cordifolia* is not deep enough. Most studies on pharmacological activities of *R. cordifolia* or its compounds are *in vitro* cell levels. Whether they have therapeutic effects on some animal models are still unclear. Besides, nearly all of the mechanism research of *R. cordifolia* or its compounds are not sufficient. Due to multiple components, targets and effects of *R. cordifolia*, it is difficult to elucidate its mechanism of action. Network pharmacology is an interdisciplinary science based on system biology, network science, bioinformatics and other related disciplines, which can clarify the relationship between drug, disease and drug targets. Using network pharmacology and experimental verifications, we explored the therapeutic effects of *Sanguisorba Offcinalis* L. a TCM against leukopenia, identified its active compounds, explored the targets of these active compounds, and illuminated the signal pathways regulated by *Sanguisorba Offcinalis* L. (Wang et al., 2020b). Network pharmacology are also specialized in illustrating the mode of action of Chinese herbal compound prescription. Our previous study demonstrated the therapeutic mechanisms underlying Beimu-Gualou Formula for the treatment of bronchiectasis using network pharmacology methods. (Shen et al., 2021). Thereby, combination of network pharmacology, high throughput sequencing including transcriptomics, proteomics, metabolomics and intestinal flora sequencing, as well as experimental verifications can deeply elucidate the effects of *R. cordifolia* or *R. cordifolia* related prescriptions on gene and protein expression, metabolites and gut microbiota changes in bodies. Another research core is to identify the direct targets of compounds derived from *R. cordifolia* against diseases. For this purpose, some technologies for identification and confirmation of drug targets should be carried out. For example, our team built a novel high-throughput screening method that combination use of biolayer interferometry (BLI) and ultra-high-performance liquid chromatography coupled with diode-array detector and quadrupole/time-of-flight tandem mass spectrometry (UHPLC−DAD-Q/TOF-MS/MS) to rapidly and effectively discover the small-molecules with amyloid-β (Aβ) binding affinity from natural medicines (Guo et al., 2022). This study strategy can be also applied to explore *R. cordifolia*.

Fourthly, most pharmacological studies of *R. cordifolia* and its active compounds lack of positive control, which reduces reliability of the data. Scientific researchs are always performed with controls to obtain reliable results. The experimental results can be critically compared, analyzed and explained with reference to the control treatments. The posive control is an experimental control that shows an experiment is working as intended and gives a positive result at the end of the experiment. If the positive control do not show the expected result, it means the design of the experiment is deficient. Therefore, further study about pharmacological activities of *R. cordifolia* and its active compounds should designs the posive control to insure the experimental results scientific, credible and solid.

Finally, the pharmacokinetics study and clinical research are extremely insufficient. *Via* pharmacokinetics study, we can confirm the medication regimen, predict the clinical efficacy and toxicity of the drugs, and provide patients with rational drug use. The shortage of pharmacokinetics study of *R. cordifolia* largely limits its clinical translation. Although *R. cordifolia* has long been used in clinic for treating different diseases in China, its clinical research is also seriously inadequate. Clinical research is a key step for clinical application of a new drug from the laboratory. The only two clinical research of *R. cordifolia* are evaluation of therapeutic effects of *R. cordifolia* on abnormal uterine bleeding, which are come from two different hospitals and the findings are published in Chinese periodicals. Others clinical data of *R. cordifolia* are not detailed record and published. We hope this herb with ancient history can be brought to the forefront by more researchers, the clinical research can be promoted and recorded, and the data can be published, which are all conducive to boost development of modernization of TCM.

In summary, this article provides a comprehensive review of the traditional applications, chemical compounds, and pharmacological activities of *R. cordifolia*. Further studies are still needed to widely identify the active compounds of *R. cordifolia*, deeply explore the pharmacological mechanisms of *R. cordifolia* and its compounds against different diseases. Meanwhile, the toxicity, pharmacokinetics and clinical research should be the focus of future investigations. We hope this review can provide some interesting information and useful suggestions for further research on TCM.
