# Supplementary material for: A comprehensive review of Rubia cordifolia L.: Traditional uses, phytochemistry, pharmacological activities, and clinical applications
Source: Front Pharmacol. 2022 Sep 9;13:965390. doi: 10.3389/fphar.2022.965390 (PMC9500525; doi:10.3389/fphar.2022.965390)
Supplement: Supplementary file 1 [file Table1.docx]

| **TABLE S1 The reported compound classification, compound name, chemical structure and molecular formula of *R. cordifolia.*** | | | | | |
| --- | --- | --- | --- | --- | --- |
| **Classification** | **NO** | **Compound name** | **Chemical structure** | **Molecular formula** | **Reference** |
| **Anthraquinones** | | | | | |
|  | 1 | Alizarin |  | C_14_H_8_O_4_ | (Wang et al., 2020a; Balachandran et al., 2021; Xu et al., 2019) |
|  | 2 | Alizarin 2-methyl ether |  | C_15_H_10_O_4_ | (Son et al., 2008) |
|  | 3 | 1-Acetoxy-3-methoxy-9,10- anthraquinone |  | C_17_H_12_O_5_ | (Son et al., 2008) |
|  | 4 | 1-Acetoxy-9,10-anthraquinone |  | C_16_H_10_O_4_ | (Son et al., 2008) |
|  | 5 | 1,3-Dihydroxy-2- methyl-9,10-anthracenedione |  | C_15_H_12_O_4_ | (Wang et al., 2020a) |
|  | 6 | 1,3-Dimethoxy-2-carboxylanthraquinone |  | C_17_H_12_O_6_ | (Dosseh et al., 1981b) |
|  | 7 | 1,4-Dihydroxy-2-methylanthraquinone |  | C_15_H_10_O_4_ | (Dosseh et al., 1981b; Singh, 2005) |
|  | 8 | 1,5-Dihydroxy-2-methylanthraquinone |  | C_15_H_10_O_4_ | (Dosseh et al., 1981b) |
|  | 9 | 1-Hydroxy-2-methyl-9,10-anthracenedione |  | C_15_H_13_O_3_ | (Wang et al., 2020a) |
|  | 10 | 6-Hydroxyrubiadin |  | C_15_H_12_O_4_ | (Wang et al., 2020a) |
|  | 11 | 1-Hydroxy-2-methylanthraquinone |  | C_15_H_10_O_3_ | (Tessier et al., 1981) |
|  | 12 | 1-Hydroxy-2-methyoxyanthraquinone |  | C_15_H_10_O_4_ | (Dosseh et al., 1981a) |
|  | 13 | 1-Hydroxy-2-methyl-9,10-anthraquinone |  | C_15_H_10_O_3_ | (Qiao et al.,1990) |
|  | 14 | 1-Hydroxy-9,10-anthraquinone |  | C_14_H_8_O_3_ | (Wang et al.,1992) |
|  | 15 | 1-Hydroxy-3-hydroxymethylanthraquinone |  | C_15_H_10_O_~~4~~_ | (Hideji et al., 1983) |
|  | 16 | 1-Hydroxy-2-hydroxymethylanthraquinone |  | C_15_H_10_O_4_ | (Itokawa et al., 1993) |
|  | 17 | 2-Hydroxy-6-methylanthraquinone |  | C_15_H_10_O_3_ | (Akhtar etal., 2006) |
|  | 18 | 2-Methyl-9,10-anthracenedione |  | C_15_H_12_O_2_ | (Wang et al., 2020a) |
|  | 19 | Nordamnacanthal |  | C_15_H_8_O_5_ | (Tessier et al., 1981) |
|  | 20 | Physcion |  | C_16_H_12_O_5_ | (Tessier et al., 1981) |
|  | 21 | Purpurin |  | C_14_H_8_O_5_ | (Wang et al., 2020a; Singh et al., 2020) |
|  | 22 | Rubiadin |  | C_15_H_10_O_4_ | (Rao et al., 2006; Watroly et al., 2021) |
|  | 23 | Rubiacordone A |  | C_23_H_22_O_10_ | (Li et al., 2009) |
|  | 24 | Soranjidiol |  | C_15_H_10_O_4_ | (Son et al., 2008) |
|  | 25 | Tectoquinone |  | C_15_H_10_O_2_ | (Lu et al., 2010) |
|  | 26 | 1,2,4,6-Tetrahydroxyanthraquinone |  | C_14_H_8_O_5_ | (Chen et al., 2018) |
|  | 27 | 1,3,6-Tirhydroxy-2-methylanthraquinone-3-*O*-α-L-rhamnosyl-(1→2)-*β*-D-glucoside |  | C_27_H_30_O_14_ | (Wang et al., 2020a) |
|  | 28 | Xanthopurpurin |  | C_14_H_8_O_4_ | (Wang et al., 2020a; Chen et al., 2017) |
| **Bicyclic hexapeptides** | | | | | |
|  | 29 | Allo-RA-V |  | C_40_H_48_N_6_O_9_ | (Hitotsuyanagi et al., 2012) |
|  | 30 | Neo-RA-V |  | C_40_H_48_N_6_O_9_ | (Hitotsuyanagi et al., 2012) |
|  | 31 | *O*-Seco-RA-V |  | C_40_H_50_N_6_O_9_ | (Hitotsuyanagi et al., 2012) |
|  | 32 | RA-ⅩⅧ |  | C_41_H_50_N_6_O_10_ | (Lee et al., 2008a) |
|  | 33 | RA-XIII |  | C_48_H_60_N_6_O_16_ | (Morita et al., 1992) |
|  | 34 | RA-XI |  | C_42_H_50_N_6_O_11_ | (Morita et al., 1992) |
|  | 35 | RA-XII |  | C_46_H_58_N_6_O_14_ | (Morita et al., 1992; Lee et al., 2008a) |
|  | 36 | RA-XIV |  | C_48_H_58_N_6_O_15_ | (Morita et al., 1992) |
|  | 37 | RA-X |  | C_43_H_52_N_6_O_11_ | ( Itokawa et al., 1992) |
|  | 38 | RA-XV |  | C_48_H_60_N_6_O_15_ | (Takeya et al., 1993) |
|  | 39 | RA-XVI |  | C_48_H_60_N_6_O_16_ | (Takeya et al., 1993) |
|  | 40 | RA-VII |  | C_41_H_50_N_6_O_9_ | (Hitotsuyanagi et al., 2016; Itokawa et al., 1992) |
|  | 41 | RA-ⅩⅩ |  | C_42_H_52_N_6_O_9_ | (Hitotsuyanagi et al., 2019) |
|  | 42 | RA-ⅩⅩⅢ |  | C_43_H_53_N_7_O_10_ | (Lee et al., 2008b) |
|  | 43 | RA-ⅩⅩⅣ |  | C_42_H_51_N_7_O_10_ | (Lee et al., 2008b; Hitotsuyanagi et al., 2019) |
|  | 44 | RA-Ⅵ |  | C_41_H_50_N_6_O_10_ | (Itokawa et al., 1991) |
|  | 45 | RA-ⅥII |  | C_42_H_52_N_6_O_10_ | (Itokawa et al., 1991) |
|  | 46 | RA-ⅩⅩⅤ |  | C_40_H_48_N_6_O_9_ | (Hitotsuyanagi et al., 2019) |
|  | 47 | RA-ⅩⅩⅥ |  | C_39_H_46_N_6_O_9_ | (Hitotsuyanagi et al., 2019) |
| **Naphthoquinones** | | | | | |
|  | 48 | Acetylmollugin |  | C_19_H_18_O_4_ | (Ho et al., 1996) |
|  | 49 | 2-Carbomethoxy-2,3-epoxy-3-prenyl-1,4-naphthoquinone |  | C_17_H_17_O_5_ | (Son et al., 2008; Jun et al., 2011) |
|  | 50 | Dihydromollugin |  | C_17_H_18_O_4_ | (Hua et al., 1992) |
|  | 51 | 1’,2’-Dihydroxydithydromollugin |  | C_17_H_18_O_6_ | (Hideji et al., 1993) |
|  | 52 | Dehydro-α-lapachone |  | C_15_H_12_O_3_ | (Wang et al., 2020a) |
|  | 53 | Epoxymollugin |  | C_17_H_16_O_5_ | (Son et al., 2008) |
|  | 54 | Furomollugin |  | C_14_H_10_O_4_ | (Son et al., 2008; Ho et al., 1996) |
|  | 55 | 2’-Hydroxymollugin |  | C_17_H_16_O_5_ | (Hideji et al., 1993; Lu et al., 2010) |
|  | 56 | Lapachol |  | C_15_H_14_O_3_ | (Wang et al., 2020a) |
|  | 57 | Mollugin |  | C_17_H_16_O_4_ | (Wang et al., 2017) |
|  | 58 | 2’-Methoxymollugin |  | C_18_H_18_O_5_ | (Hideji et al., 1993) |
|  | 59 | 1’-Methoxy-2’-hydroxydithydromollugin |  | C_18_H_20_O_6_ | (Hideji et al., 1993) |
|  | 60 | 3-Prenyl-5-methoxynaphthoquinone |  | C1_6_H_16_O_3_ | (Dosseh et al., 1981b) |
|  | 61 | 3-Prenyl-8-methoxynaphthoquinone |  | C_16_H_16_O_3_ | (Dosseh et al., 1981b) |
|  | 62 | Rubilactone |  | C_15_H_10_O_5_ | (Ho et al., 1996) |
|  | 63 | Rubioncolin |  | C_27_H_21_O_6_ | (Wang et al., 2020a) |
| **Triterpenoids** | | | | | |
|  | 64 | 3-*β*-Friedelinol |  | C_30_H_52_O_6_ | (Ibraheim et al., 2010) |
|  | 65 | Oleanolic acid |  | C_30_H_48_O_3_ | (Son et al., 2008) |
|  | 66 | Rubicoumaric acid |  | C_39_H_54_O_6_ | (Talapatra et al., 1981) |
|  | 67 | Rubifolic acid |  | C_30_H_48_O_4_ | (Talapatra et al., 1981) |
|  | 68 | Rubiprasin A |  | C_32_H_52_O_5_ | (Itokawa et al., 1989a) |
|  | 69 | Rubiarbonol A |  | C_30_H_50_O_4_ | (Son et al., 2006) |
|  | 70 | Rubiarbonol B |  | C_30_H_50_O_3_ | (Son et al., 2006) |
|  | 71 | Ursolic acid |  | C_30_H_48_O_3_ | (Wang,2014) |
| **Other compounds** | | | | | |
|  | 72 | Atraric acid |  | C_10_H_12_O_4_ | (Ibraheim et al., 2010) |
|  | 73 | 3,3’-Bis(3,4-dihydro-4-hydroxy-6-methoxy-2h-1-benzopyran) |  | C_20_H_22_O_6_ | (Son et al., 2008) |
|  | 74 | D-3-*O*-methoxychiroinositol |  | C_7_H_14_O_6_ | (Ibraheim et al., 2010) |
|  | 75 | Daucosterol |  | C_35_H_60_O_6_ | (Qiao et al.,1990) |
|  | 76 | 3,5-Di-(phydroxybenzyl)phenol |  | C_8_H_10_O_3_ | (Akhtar et al., 2006) |
|  | 77 | 2,3-Dihydro-2-(4-hydroxy-3-methoxyphenyl)-3-hydroxymethy-1-5-w-hydroxyl7-methoxybenzofuran |  | C_20_H_24_O_6_ | (Son et al., 2006; Jeong et al., 2012) |
|  | 78 | 8-Hydroxy n-pentadecanyl decan-4-en-1-oate |  | C_25_H_48_O_3_ | (Akhtar et al., 2006) |
|  | 79 | (+)-Laricirestnol |  | C_24_H_24_O_6_ | (Lu et al., 2010) |
|  | 80 | N-heptadecane |  | C_17_H_36_ | (Akhtar et al., 2006) |
|  | 81 | N-octacosanyl octa-1-oate |  | C_36_H_72_O_2_ | (Akhtar et al., 2006) |
|  | 82 | N-nonadecane |  | C_19_H_40_ | (Akhtar et al., 2006) |
|  | 83 | Palmitic acid |  | C_16_H_32_O_2_ | (Wang, 2014) |
|  | 84 | Rubilactone |  | C_15_H_10_O_5_ | (Ho et al., 1996; Hassanean et al., 2000) |
|  | 85 | Rubiasin A |  | C_15_H_16_O_2_ | (Lu et al., 2010) |
|  | 86 | *β*-Sitosterol |  | C_29_H_50_O | (Son et al., 2006 ; Qiao et al.,1990) |
|  | 87 | Tricosanoic acid |  | C_23_H_46_O_2_ | (Wang, 2014) |
|  | 88 | 1,3,6-Trihydroxy-2-methyl-9,10-anthraqueinone-3-α-L-rhamnosyl (1→2)-*β*-D-glucoside |  | C_29_H_32_O_15_ | (Qiao et al.,1990) |
|  | 89 | Vanillic acid |  | C_8_H_8_O_4_ | (Ibraheim et al., 2010) |
|  | 90 | 2-Carboxymethy-3-prenyl-2,3-epoxynaphthoquinone | Not available | C_17_H_16_O_5_ | (Itokawa et al., 1993; Ho et al., 1996) |
|  | 91 | 2-Carbomethoxy-3-(3’-hydroxy)-isopentyl-1,4-naphthohydroquinone-4-*O*-*β*-glucoside | Not available | C_23_H_30_O_10_ | (Itokawa et al., 1989b) |
|  | 92 | 2-Carbomethoxy-3-prenyl-1,4-naphthohydroquinone-1,4-di-*O*-*β*-glucoside | Not available | C_29_H_38_O_14_ | (Itokawa et al., 1989b) |
|  | 93 | 1,8-Dihydroxy-11,20 (pentylnaphthaquinone) penanthrene | Not available | C_26_H_20_O_4_ | (Abdullah et al.,2003) |
|  | 94 | 6-Hydroxy-2-(5-hydroxy-4-methoxycarbonyl-naphto [1,2,6] furan-2-yl)-2-methyl-3,4-dihydro-2h-ben20[h]-chromene-5-carboxylic acidmethy ester | Not available | C_30_H_24_O_8_ | (Hideji et al., 1993) |
|  | 95 | 5-Hydroxy-2-[7hydroxy-4-(1-hydroxy-1-methylethyl)-2-methyl-6-oxo-2,3,3a-6-tetrahydro-4h-1,5-dioxaben20-[de]anthracene-2-yl]-naphtho[1,2-b] furan-4-carboxylic acidmenty ester | Not available | C_33_H_28_O_9_ | (Hideji et al., 1993) |
|  | 96 | 1,3,6-Trihydroxy-2-methylanthraquinone-3-o-(4’,6’-o-diacethyl)-α-rhamnosyl-(1→2)-*β-*glucoside | Not available | C_31_H_34_O_16_ | (Itokawa et al., 1989b) |
|  | 97 | 1,3,6-Trihydroxy-2-methylanthraquinone-3-*O*-(6’-*O*-acetyl)-α-ramnosyl-(1→2)-*β*-glucoside | Not available | C_29_H_32_O_15_ | (Itokawa et al., 1989b) |
|  | 98 | 1,3,6-Tirhydroxy-2-methylanthraquinone-3-*O*-(3’,6’-*O*-diacetyl)-α-rhamnosyl-(1→2)-*β*-glucoside | Not available | C_31_H_34_O_16_ | (Itokawa et al., 1989b) |
| RA: Rubiaakane, O: Oxygen. | | | | | |
